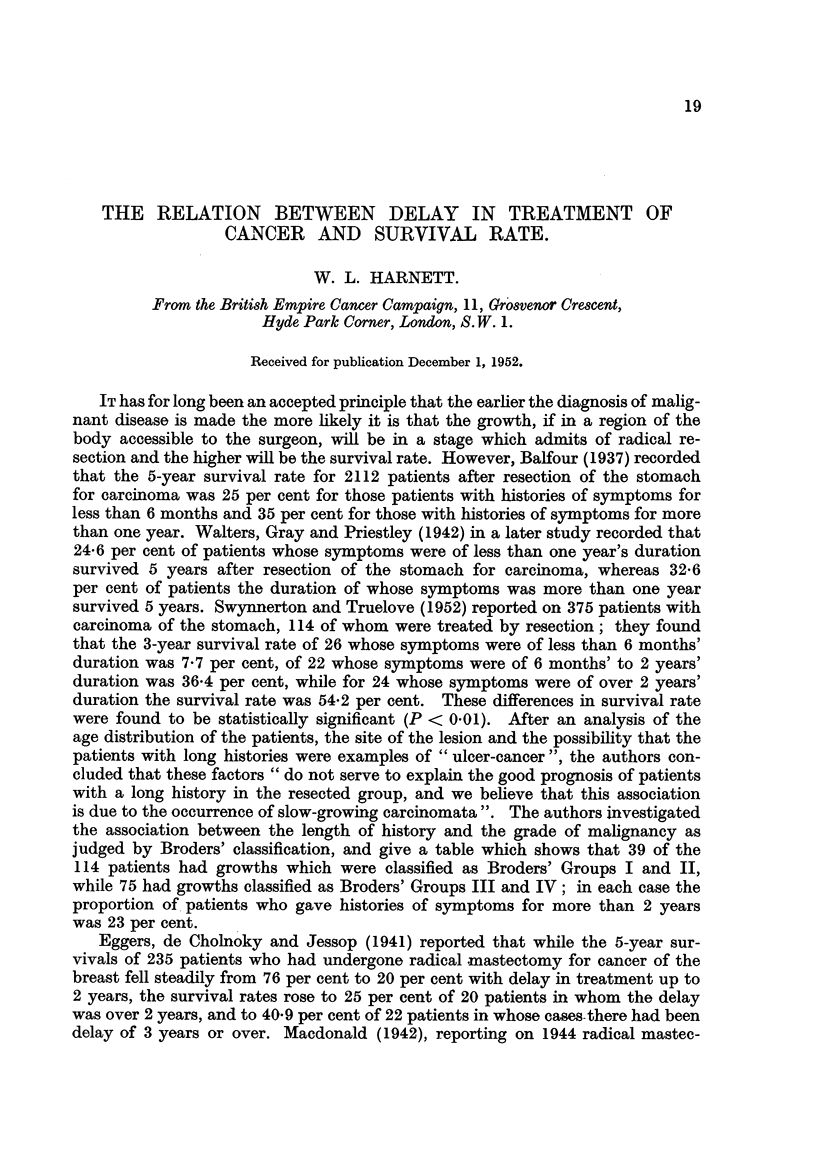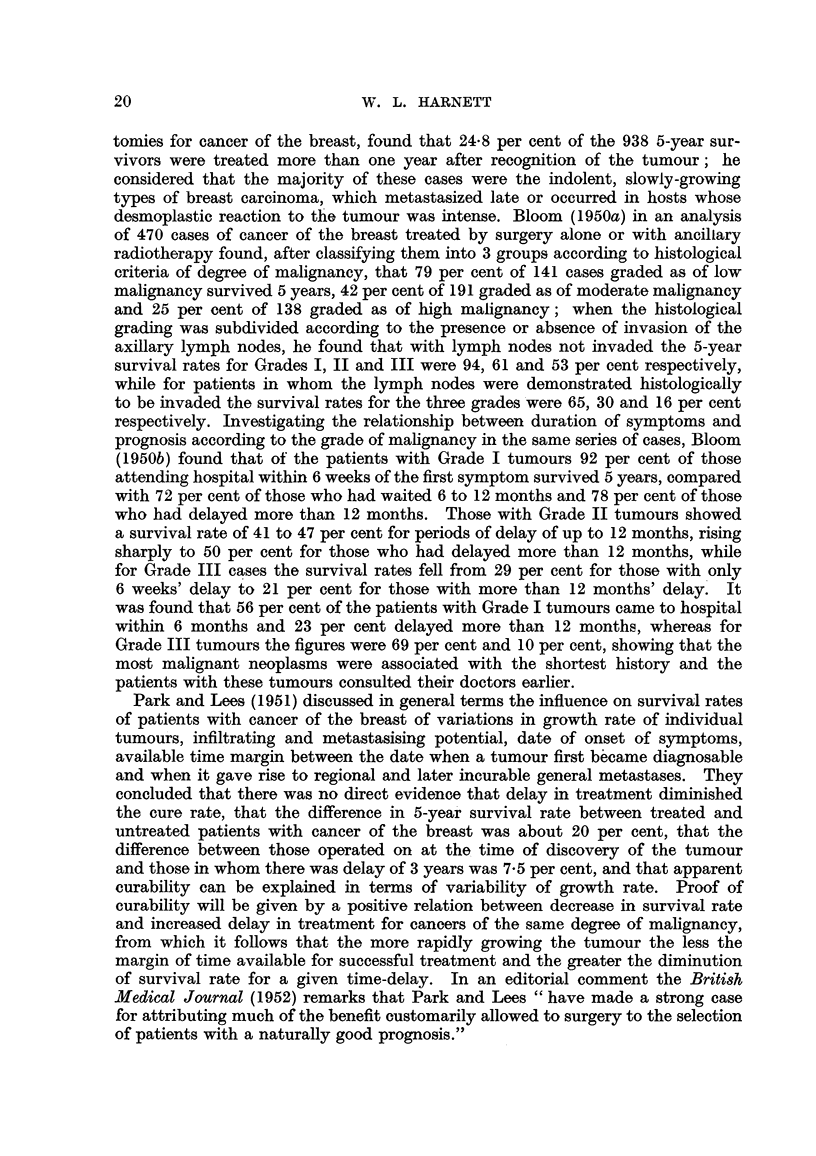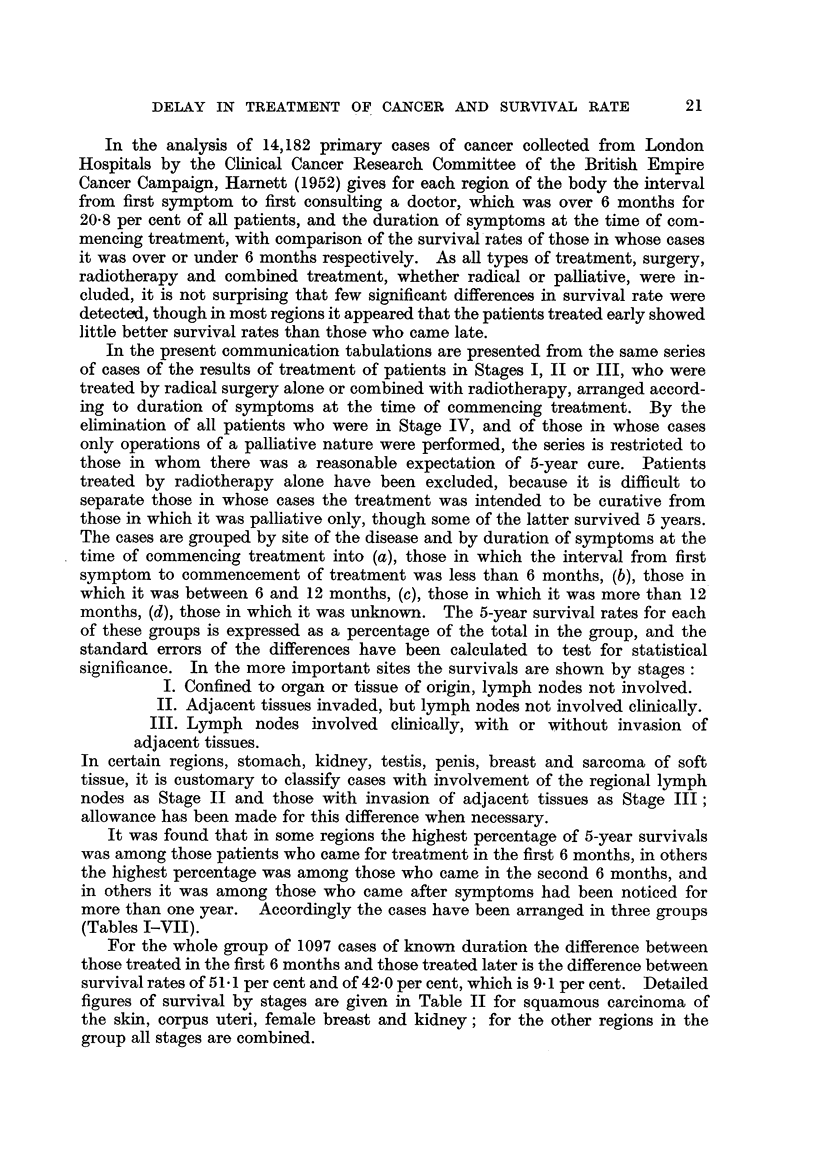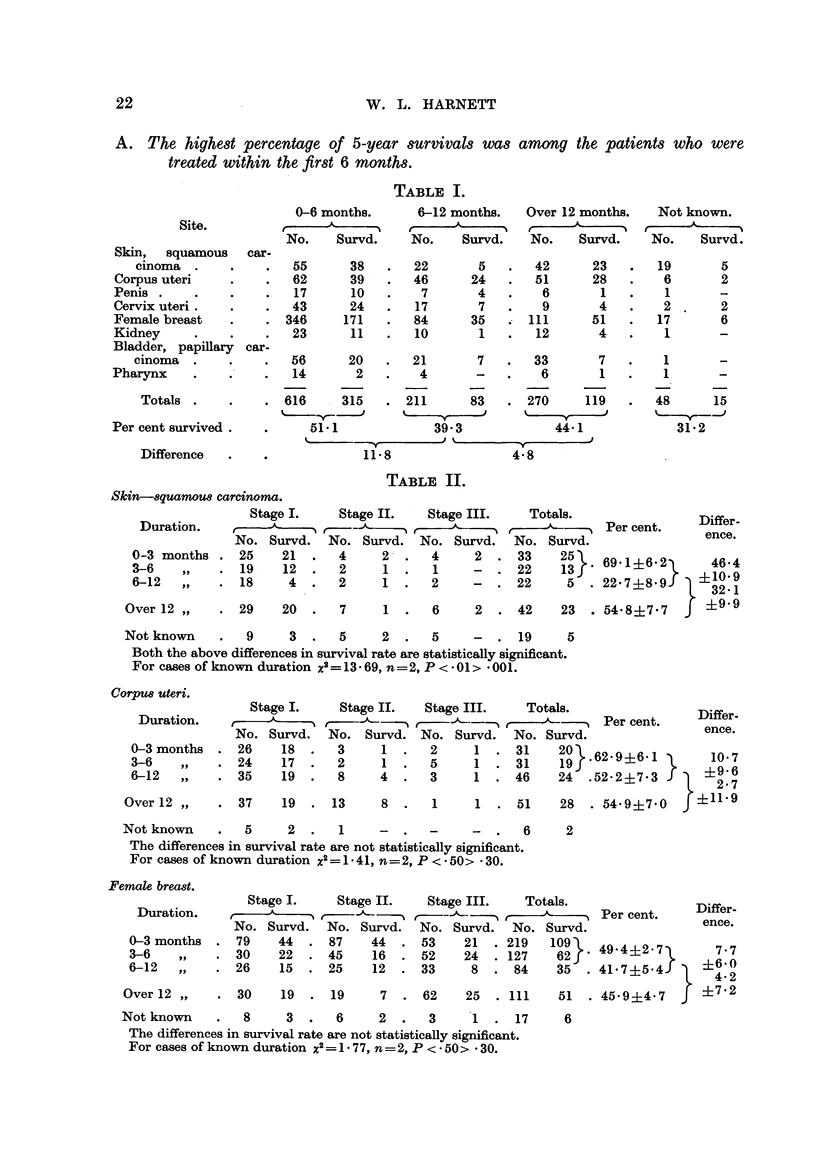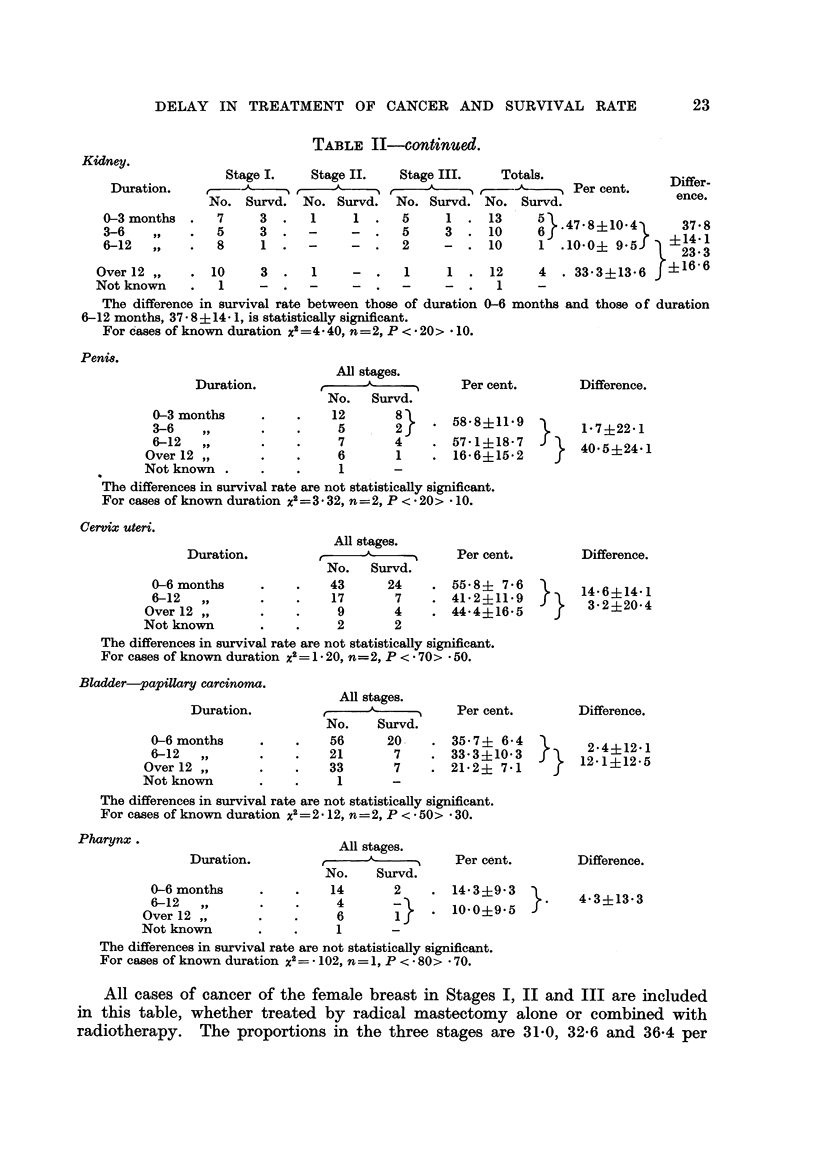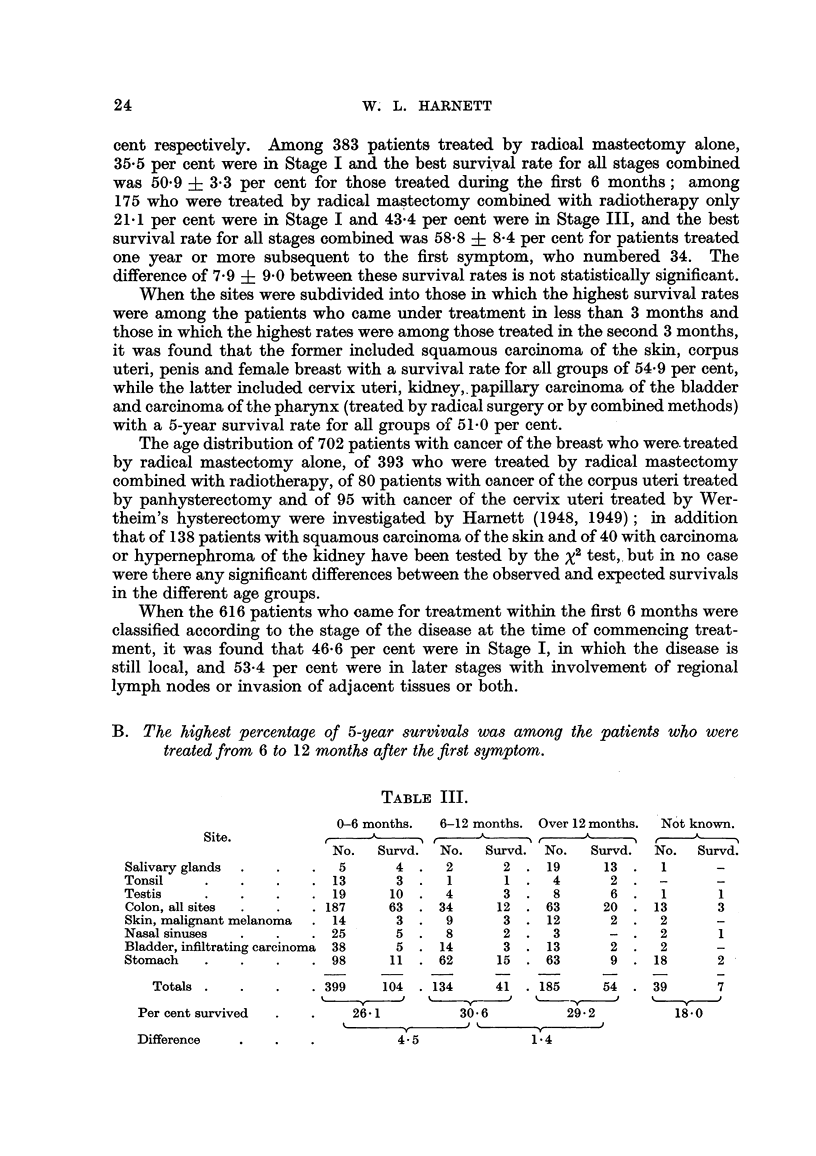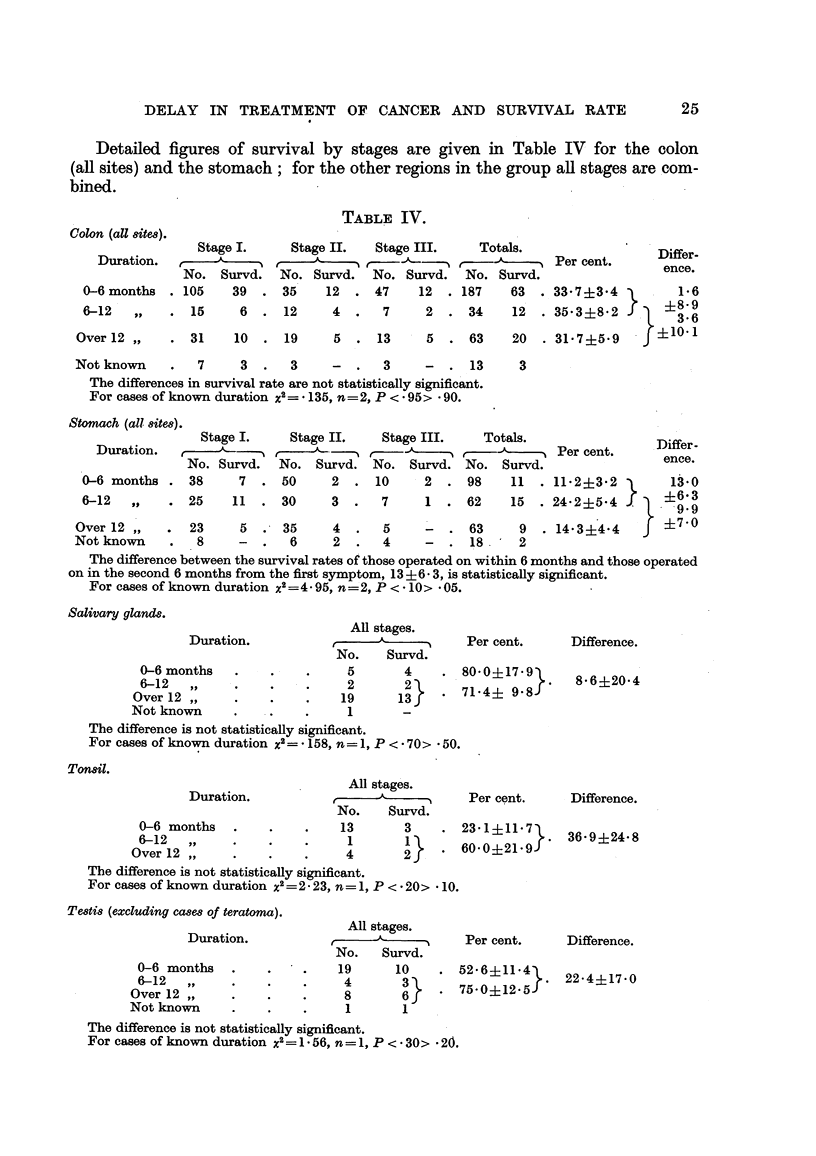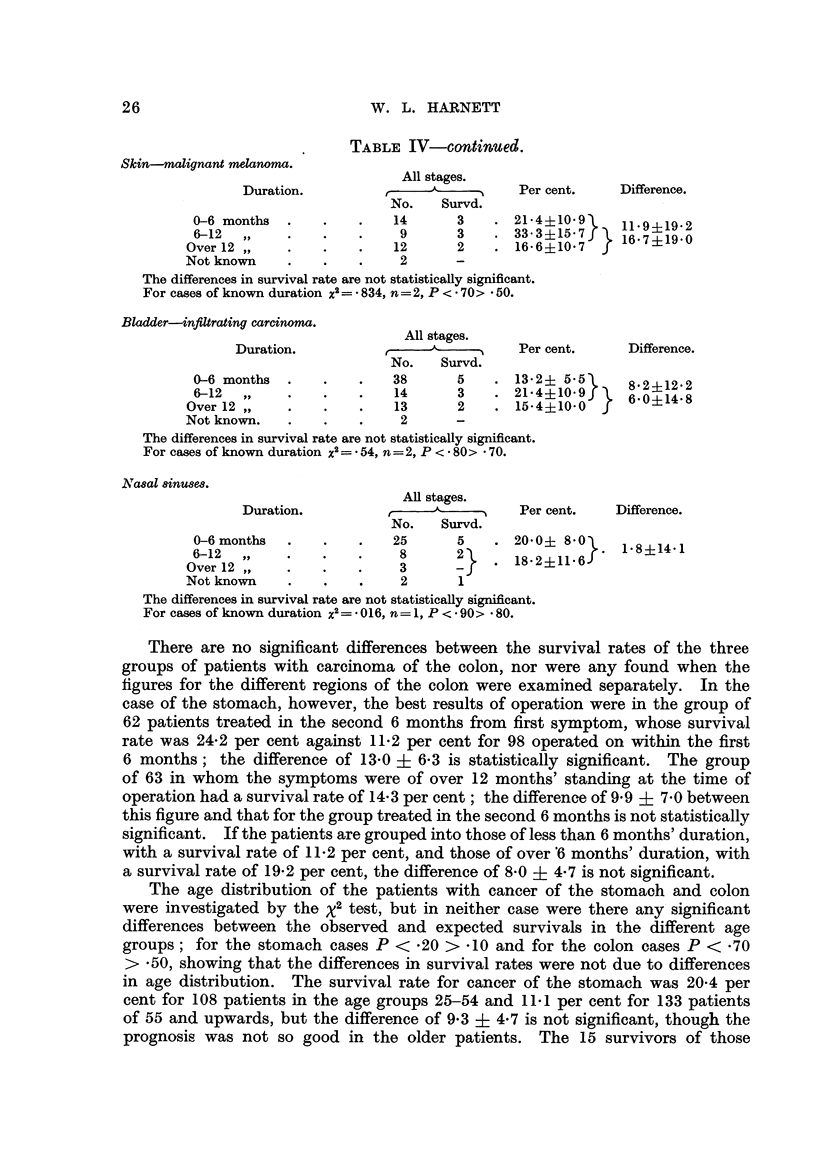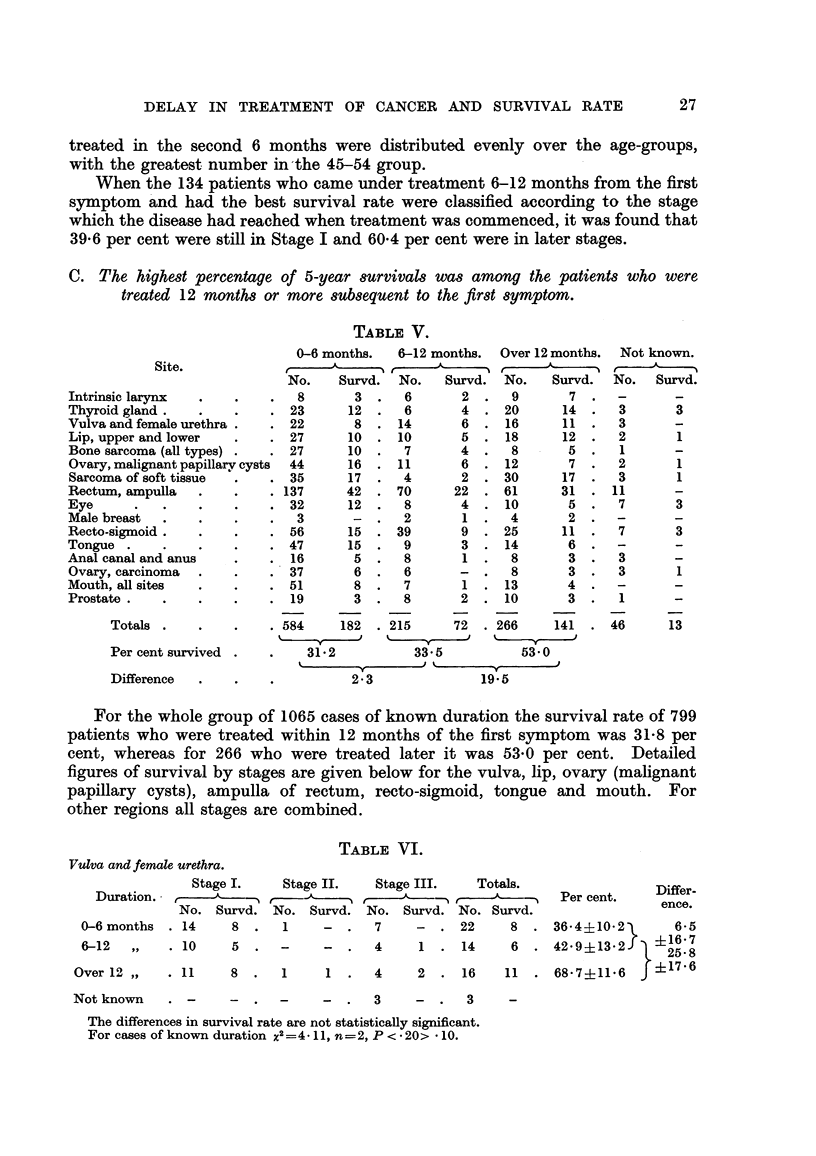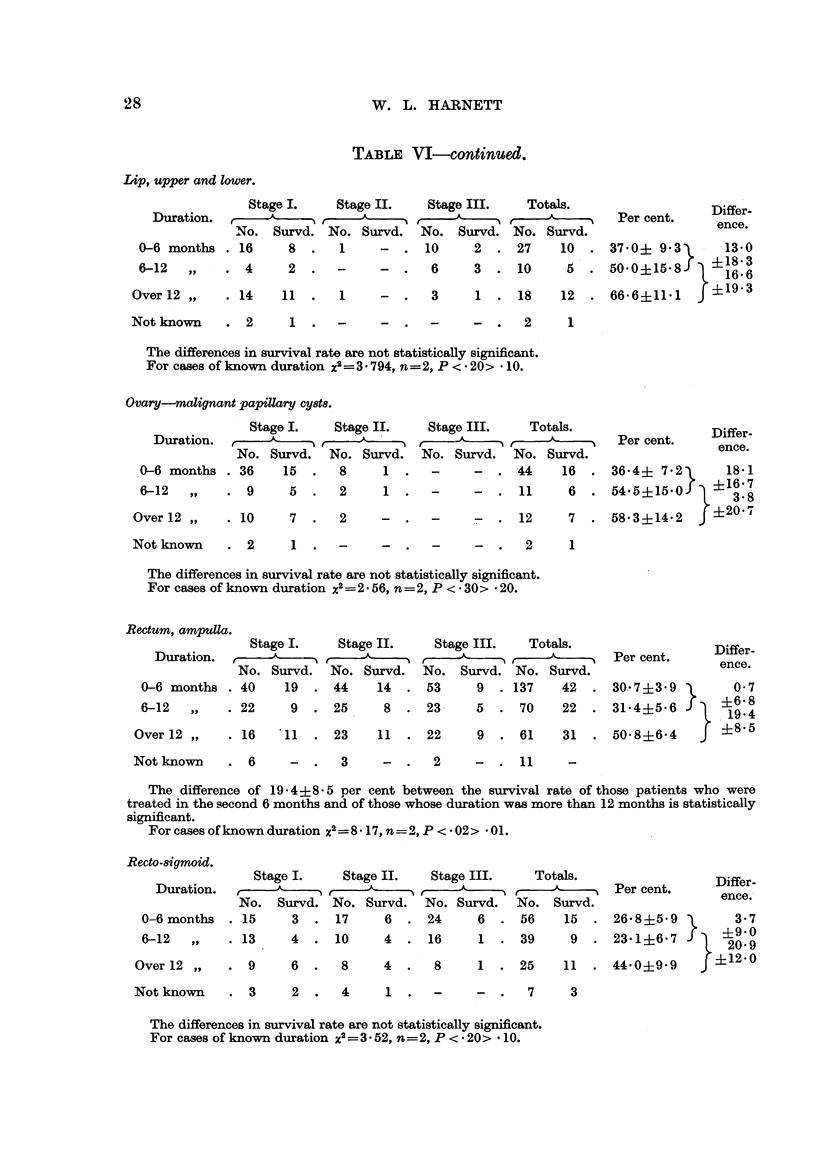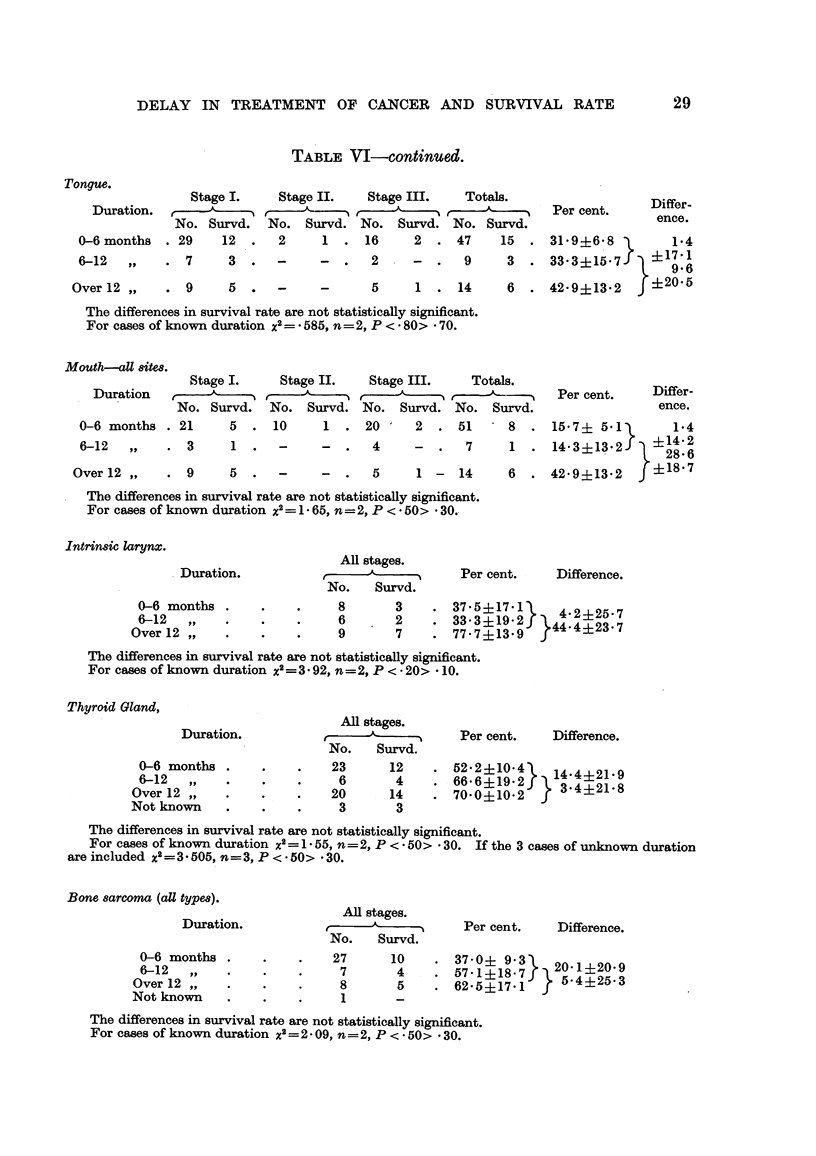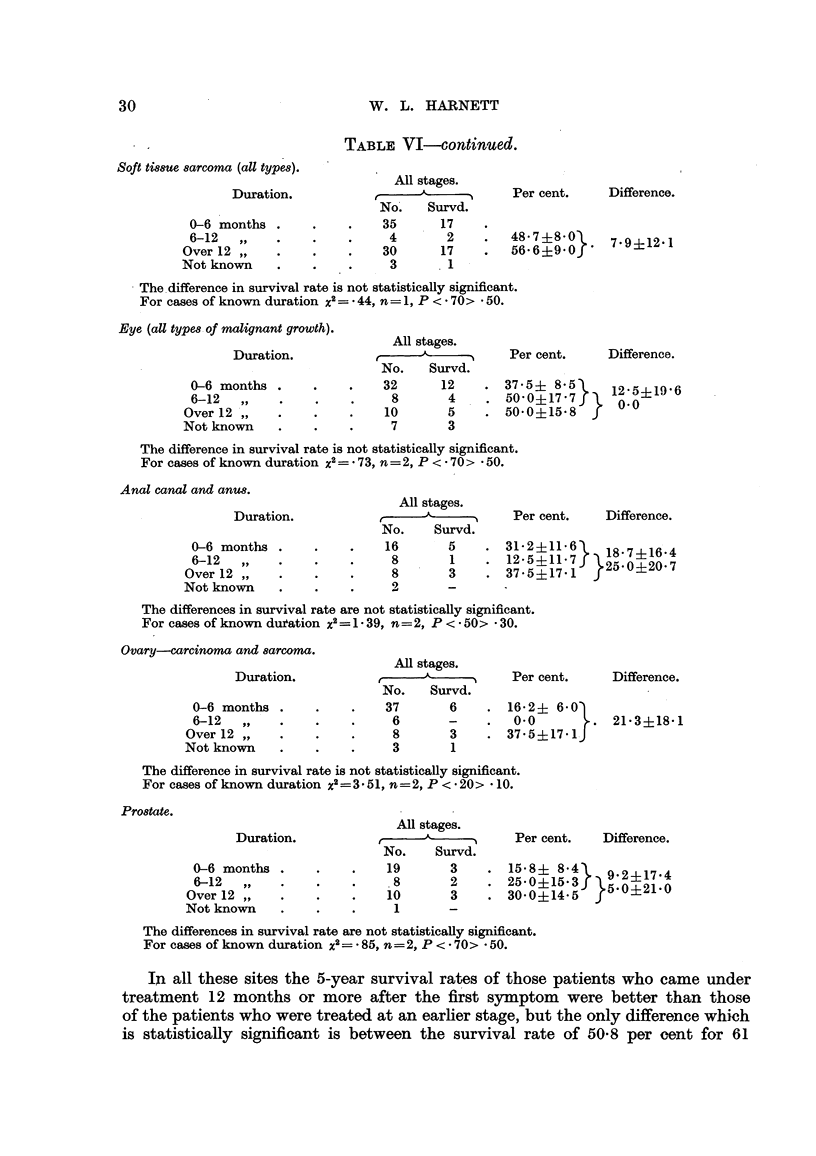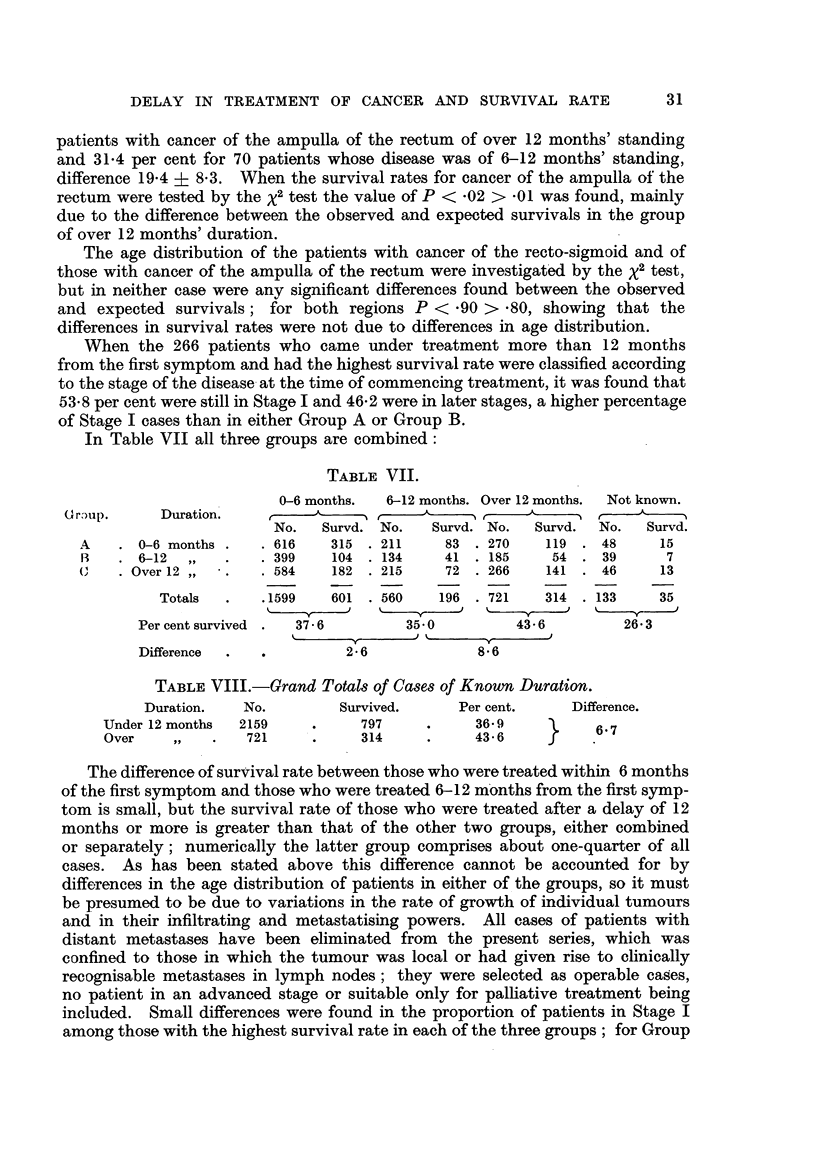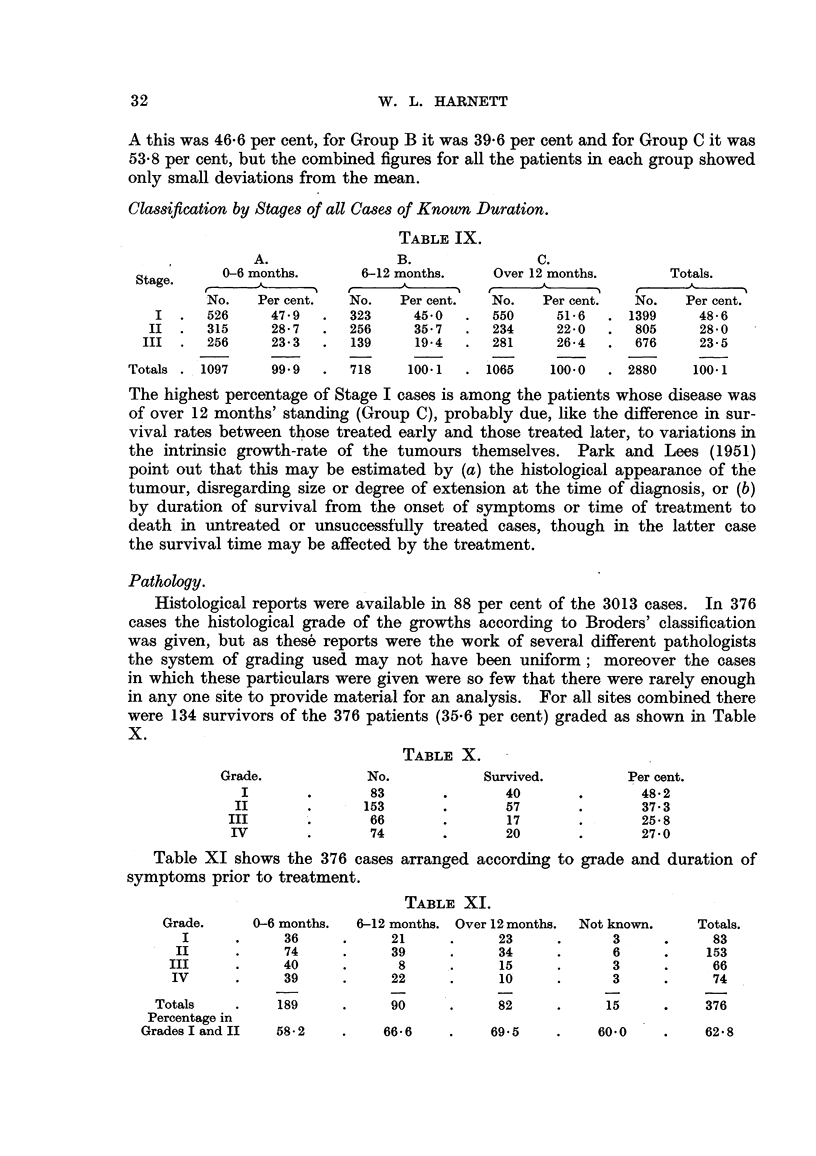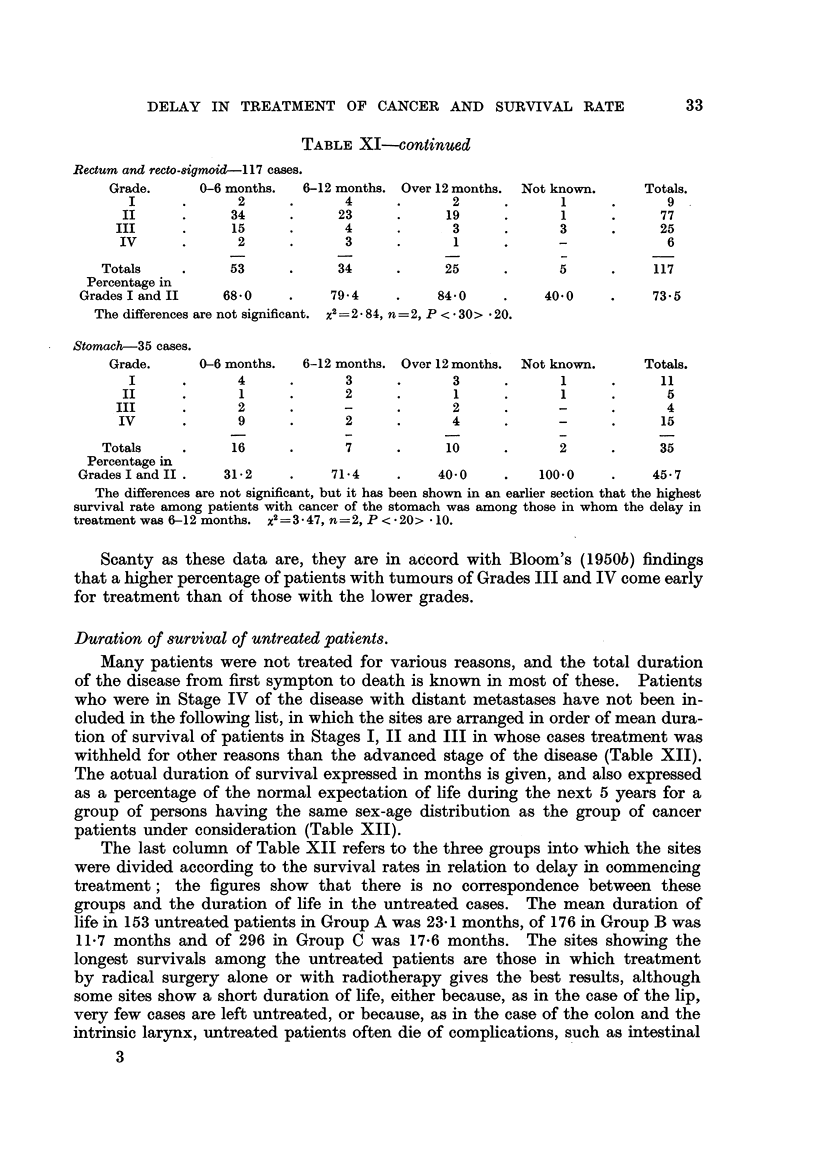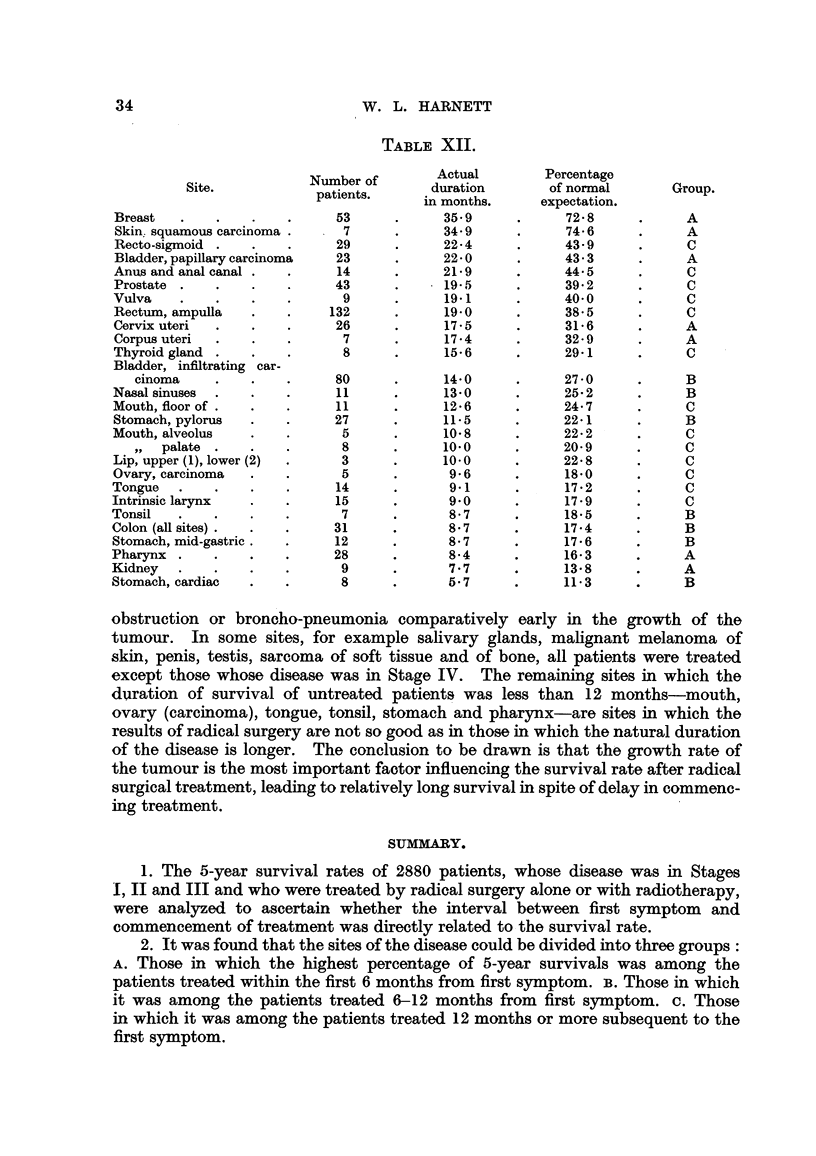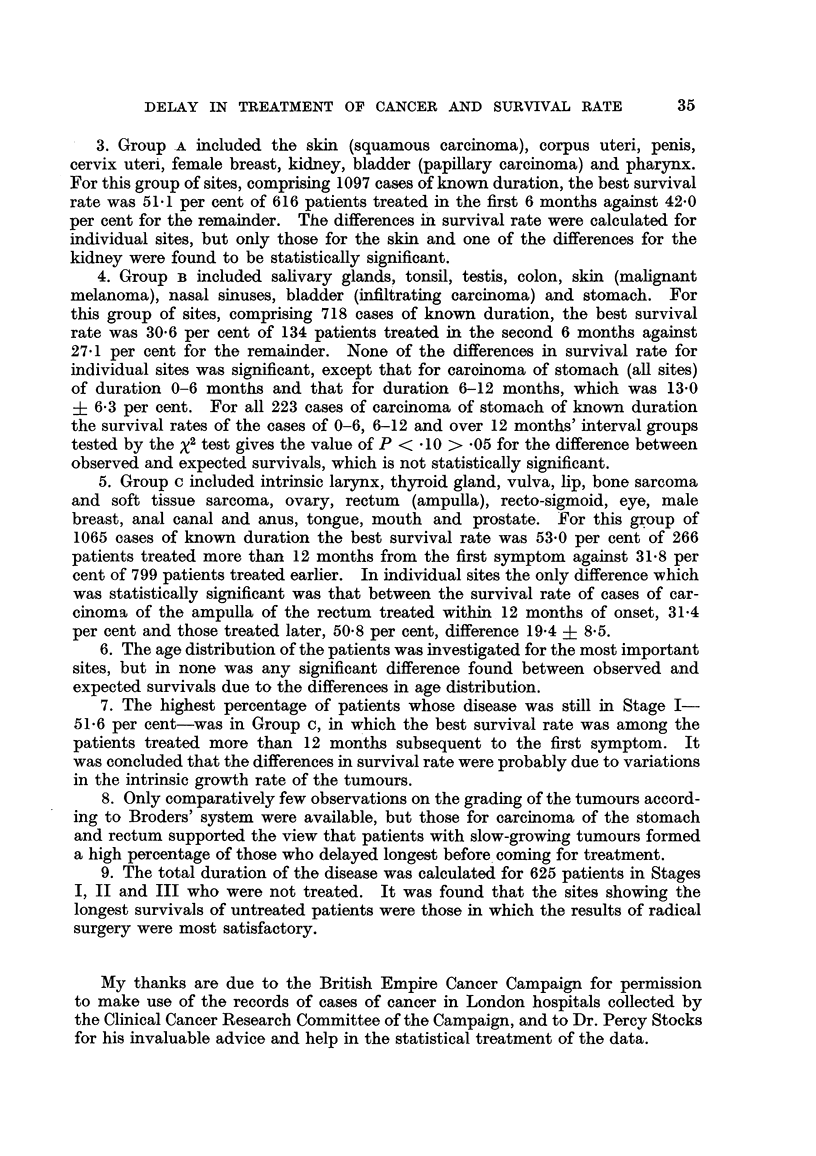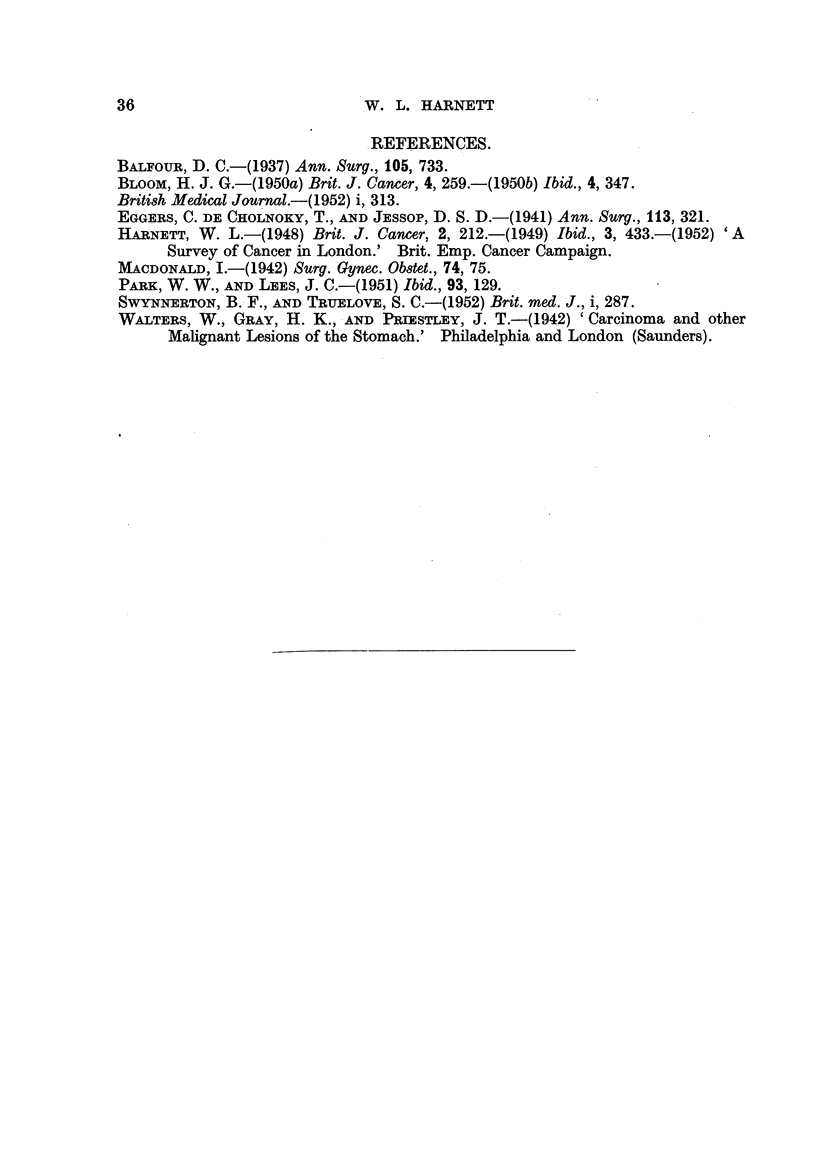# The Relation Between Delay in Treatment of Cancer and Survival Rate

**DOI:** 10.1038/bjc.1953.3

**Published:** 1953-03

**Authors:** W. L. Harnett


### THE RELATION BETWEEN DELAY IN TREATMENT OF CANCER AND SURVIVAL RATE.

W. L. HARNETT.

*From the British Empire Cancer Campaign, 11, Grosvenor Crescent, Hyde Park Corner, London, S.W. 1.*

Received for publication December 1, 1952.

It has for long been an accepted principle that the earlier the diagnosis of malignant disease is made the more likely it is that the growth, if in a region of the body accessible to the surgeon, will be in a stage which admits of radical resection and the higher will be the survival rate. However, Balfour (1937) recorded that the 5-year survival rate for 2112 patients after resection of the stomach for carcinoma was 25 per cent for those patients with histories of symptoms for less than 6 months and 35 per cent for those with histories of symptoms for more than one year. Walters, Gray and Priestley (1942) in a later study recorded that 24·6 per cent of patients whose symptoms were of less than one year's duration survived 5 years after resection of the stomach for carcinoma, whereas 32·6 per cent of patients the duration of whose symptoms was more than one year survived 5 years. Swynnerton and Truelove (1952) reported on 375 patients with carcinoma of the stomach, 114 of whom were treated by resection ; they found that the 3-year survival rate of 26 whose symptoms were of less than 6 months' duration was 7·7 per cent, of 22 whose symptoms were of 6 months' to 2 years' duration was 36·4 per cent, while for 24 whose symptoms were of over 2 years' duration the survival rate was 54·2 per cent. These differences in survival rate were found to be statistically significant ($P < 0{\cdot}01$). After an analysis of the age distribution of the patients, the site of the lesion and the possibility that the patients with long histories were examples of " ulcer-cancer ", the authors concluded that these factors " do not serve to explain the good prognosis of patients with a long history in the resected group, and we believe that this association is due to the occurrence of slow-growing carcinomata ". The authors investigated the association between the length of history and the grade of malignancy as judged by Broders' classification, and give a table which shows that 39 of the 114 patients had growths which were classified as Broders' Groups I and II, while 75 had growths classified as Broders' Groups III and IV ; in each case the proportion of patients who gave histories of symptoms for more than 2 years was 23 per cent.

Eggers, de Cholnoky and Jessop (1941) reported that while the 5-year survivals of 235 patients who had undergone radical mastectomy for cancer of the breast fell steadily from 76 per cent to 20 per cent with delay in treatment up to 2 years, the survival rates rose to 25 per cent of 20 patients in whom the delay was over 2 years, and to 40·9 per cent of 22 patients in whose cases there had been delay of 3 years or over. Macdonald (1942), reporting on 1944 radical mastectomies for cancer of the breast, found that 24·8 per cent of the 938 5-year survivors were treated more than one year after recognition of the tumour; he considered that the majority of these cases were the indolent, slowly-growing types of breast carcinoma, which metastasized late or occurred in hosts whose desmoplastic reaction to the tumour was intense. Bloom (1950*a*) in an analysis of 470 cases of cancer of the breast treated by surgery alone or with ancillary radiotherapy found, after classifying them into 3 groups according to histological criteria of degree of malignancy, that 79 per cent of 141 cases graded as of low malignancy survived 5 years, 42 per cent of 191 graded as of moderate malignancy and 25 per cent of 138 graded as of high malignancy; when the histological grading was subdivided according to the presence or absence of invasion of the axillary lymph nodes, he found that with lymph nodes not invaded the 5-year survival rates for Grades I, II and III were 94, 61 and 53 per cent respectively, while for patients in whom the lymph nodes were demonstrated histologically to be invaded the survival rates for the three grades were 65, 30 and 16 per cent respectively. Investigating the relationship between duration of symptoms and prognosis according to the grade of malignancy in the same series of cases, Bloom (1950*b*) found that of the patients with Grade I tumours 92 per cent of those attending hospital within 6 weeks of the first symptom survived 5 years, compared with 72 per cent of those who had waited 6 to 12 months and 78 per cent of those who had delayed more than 12 months. Those with Grade II tumours showed a survival rate of 41 to 47 per cent for periods of delay of up to 12 months, rising sharply to 50 per cent for those who had delayed more than 12 months, while for Grade III cases the survival rates fell from 29 per cent for those with only 6 weeks' delay to 21 per cent for those with more than 12 months' delay. It was found that 56 per cent of the patients with Grade I tumours came to hospital within 6 months and 23 per cent delayed more than 12 months, whereas for Grade III tumours the figures were 69 per cent and 10 per cent, showing that the most malignant neoplasms were associated with the shortest history and the patients with these tumours consulted their doctors earlier.

Park and Lees (1951) discussed in general terms the influence on survival rates of patients with cancer of the breast of variations in growth rate of individual tumours, infiltrating and metastasising potential, date of onset of symptoms, available time margin between the date when a tumour first became diagnosable and when it gave rise to regional and later incurable general metastases. They concluded that there was no direct evidence that delay in treatment diminished the cure rate, that the difference in 5-year survival rate between treated and untreated patients with cancer of the breast was about 20 per cent, that the difference between those operated on at the time of discovery of the tumour and those in whom there was delay of 3 years was 7·5 per cent, and that apparent curability can be explained in terms of variability of growth rate. Proof of curability will be given by a positive relation between decrease in survival rate and increased delay in treatment for cancers of the same degree of malignancy, from which it follows that the more rapidly growing the tumour the less the margin of time available for successful treatment and the greater the diminution of survival rate for a given time-delay. In an editorial comment the *British Medical Journal* (1952) remarks that Park and Lees "have made a strong case for attributing much of the benefit customarily allowed to surgery to the selection of patients with a naturally good prognosis."

In the analysis of 14,182 primary cases of cancer collected from London Hospitals by the Clinical Cancer Research Committee of the British Empire Cancer Campaign, Harnett (1952) gives for each region of the body the interval from first symptom to first consulting a doctor, which was over 6 months for 20·8 per cent of all patients, and the duration of symptoms at the time of commencing treatment, with comparison of the survival rates of those in whose cases it was over or under 6 months respectively. As all types of treatment, surgery, radiotherapy and combined treatment, whether radical or palliative, were included, it is not surprising that few significant differences in survival rate were detected, though in most regions it appeared that the patients treated early showed little better survival rates than those who came late.

In the present communication tabulations are presented from the same series of cases of the results of treatment of patients in Stages I, II or III, who were treated by radical surgery alone or combined with radiotherapy, arranged according to duration of symptoms at the time of commencing treatment. By the elimination of all patients who were in Stage IV, and of those in whose cases only operations of a palliative nature were performed, the series is restricted to those in whom there was a reasonable expectation of 5-year cure. Patients treated by radiotherapy alone have been excluded, because it is difficult to separate those in whose cases the treatment was intended to be curative from those in which it was palliative only, though some of the latter survived 5 years. The cases are grouped by site of the disease and by duration of symptoms at the time of commencing treatment into (*a*), those in which the interval from first symptom to commencement of treatment was less than 6 months, (*b*), those in which it was between 6 and 12 months, (*c*), those in which it was more than 12 months, (*d*), those in which it was unknown. The 5-year survival rates for each of these groups is expressed as a percentage of the total in the group, and the standard errors of the differences have been calculated to test for statistical significance. In the more important sites the survivals are shown by stages :

I. Confined to organ or tissue of origin, lymph nodes not involved.
II. Adjacent tissues invaded, but lymph nodes not involved clinically.
III. Lymph nodes involved clinically, with or without invasion of adjacent tissues.

In certain regions, stomach, kidney, testis, penis, breast and sarcoma of soft tissue, it is customary to classify cases with involvement of the regional lymph nodes as Stage II and those with invasion of adjacent tissues as Stage III ; allowance has been made for this difference when necessary.

It was found that in some regions the highest percentage of 5-year survivals was among those patients who came for treatment in the first 6 months, in others the highest percentage was among those who came in the second 6 months, and in others it was among those who came after symptoms had been noticed for more than one year. Accordingly the cases have been arranged in three groups (Tables I–VII).

For the whole group of 1097 cases of known duration the difference between those treated in the first 6 months and those treated later is the difference between survival rates of 51·1 per cent and of 42·0 per cent, which is 9·1 per cent. Detailed figures of survival by stages are given in Table II for squamous carcinoma of the skin, corpus uteri, female breast and kidney ; for the other regions in the group all stages are combined.

A. *The highest percentage of 5-year survivals was among the patients who were treated within the first* 6 *months.*

TABLE I.

| Site. | 0–6 months. | | 6–12 months. | | Over 12 months. | | Not known. | |
|---|---|---|---|---|---|---|---|---|
| | No. | Survd. | No. | Survd. | No. | Survd. | No. | Survd. |
| Skin, squamous carcinoma | 55 | 38 | 22 | 5 | 42 | 23 | 19 | 5 |
| Corpus uteri | 62 | 39 | 46 | 24 | 51 | 28 | 6 | 2 |
| Penis | 17 | 10 | 7 | 4 | 6 | 1 | 1 | – |
| Cervix uteri | 43 | 24 | 17 | 7 | 9 | 4 | 2 | 2 |
| Female breast | 346 | 171 | 84 | 35 | 111 | 51 | 17 | 6 |
| Kidney | 23 | 11 | 10 | 1 | 12 | 4 | 1 | – |
| Bladder, papillary carcinoma | 56 | 20 | 21 | 7 | 33 | 7 | 1 | – |
| Pharynx | 14 | 2 | 4 | – | 6 | 1 | 1 | – |
| Totals | 616 | 315 | 211 | 83 | 270 | 119 | 48 | 15 |
| Per cent survived | 51·1 | | 39·3 | | 44·1 | | 31·2 | |
| Difference | 11·8 | | 4·8 | | | | | |

TABLE II.

*Skin—squamous carcinoma.*

| Duration. | Stage I. | | Stage II. | | Stage III. | | Totals. | | Per cent. | Difference. |
|---|---|---|---|---|---|---|---|---|---|---|
| | No. | Survd. | No. | Survd. | No. | Survd. | No. | Survd. | | |
| 0-3 months | 25 | 21 | 4 | 2 | 4 | 2 | 33 | 25 | 69·1±6·2 (0–6 months) | 46·4 ±10·9 |
| 3–6 " | 19 | 12 | 2 | 1 | 1 | – | 22 | 13 | | |
| 6–12 " | 18 | 4 | 2 | 1 | 2 | – | 22 | 5 | 22·7±8·9 | 32·1 ±9·9 |
| Over 12 " | 29 | 20 | 7 | 1 | 6 | 2 | 42 | 23 | 54·8±7·7 | |
| Not known | 9 | 3 | 5 | 2 | 5 | – | 19 | 5 | | |

Both the above differences in survival rate are statistically significant.
For cases of known duration $\chi^2=13\cdot69$, $n=2$, $P<\cdot01>\cdot001$.

*Corpus uteri.*

| Duration. | Stage I. | | Stage II. | | Stage III. | | Totals. | | Per cent. | Difference. |
|---|---|---|---|---|---|---|---|---|---|---|
| | No. | Survd. | No. | Survd. | No. | Survd. | No. | Survd. | | |
| 0–3 months | 26 | 18 | 3 | 1 | 2 | 1 | 31 | 20 | 62·9±6·1 (0–6 months) | 10·7 ±9·6 |
| 3–6 " | 24 | 17 | 2 | 1 | 5 | 1 | 31 | 19 | | |
| 6–12 " | 35 | 19 | 8 | 4 | 3 | 1 | 46 | 24 | 52·2±7·3 | 2·7 ±11·9 |
| Over 12 " | 37 | 19 | 13 | 8 | 1 | 1 | 51 | 28 | 54·9±7·0 | |
| Not known | 5 | 2 | 1 | – | – | – | 6 | 2 | | |

The differences in survival rate are not statistically significant.
For cases of known duration $\chi^2=1\cdot41$, $n=2$, $P<\cdot50>\cdot30$.

*Female breast.*

| Duration. | Stage I. | | Stage II. | | Stage III. | | Totals. | | Per cent. | Difference. |
|---|---|---|---|---|---|---|---|---|---|---|
| | No. | Survd. | No. | Survd. | No. | Survd. | No. | Survd. | | |
| 0–3 months | 79 | 44 | 87 | 44 | 53 | 21 | 219 | 109 | 49·4±2·7 (0–6 months) | 7·7 ±6·0 |
| 3–6 " | 30 | 22 | 45 | 16 | 52 | 24 | 127 | 62 | | |
| 6–12 " | 26 | 15 | 25 | 12 | 33 | 8 | 84 | 35 | 41·7±5·4 | 4·2 ±7·2 |
| Over 12 " | 30 | 19 | 19 | 7 | 62 | 25 | 111 | 51 | 45·9±4·7 | |
| Not known | 8 | 3 | 6 | 2 | 3 | 1 | 17 | 6 | | |

The differences in survival rate are not statistically significant.
For cases of known duration $\chi^2=1\cdot77$, $n=2$, $P<\cdot50>\cdot30$.

TABLE II—*continued.*

*Kidney.*

| Duration. | Stage I. No. | Stage I. Survd. | Stage II. No. | Stage II. Survd. | Stage III. No. | Stage III. Survd. | Totals. No. | Totals. Survd. | Per cent. | Difference. |
|---|---|---|---|---|---|---|---|---|---|---|
| 0–3 months | 7 | 3 | 1 | 1 | 5 | 1 | 13 | 5 | } 47·8±10·4 | } 37·8 ±14·1 |
| 3–6 „ | 5 | 3 | – | – | 5 | 3 | 10 | 6 | | |
| 6–12 „ | 8 | 1 | – | – | 2 | – | 10 | 1 | 10·0± 9·5 | } 23·3 ±16·6 |
| Over 12 „ | 10 | 3 | 1 | – | 1 | 1 | 12 | 4 | 33·3±13·6 | |
| Not known | 1 | – | – | – | – | – | 1 | – | | |

The difference in survival rate between those of duration 0–6 months and those of duration 6–12 months, 37·8±14·1, is statistically significant.
For cases of known duration $\chi^2=4\cdot40$, $n=2$, $P<\cdot20>\cdot10$.

*Penis.*

| Duration. | All stages. No. | All stages. Survd. | Per cent. | Difference. |
|---|---|---|---|---|
| 0–3 months | 12 | 8 | } 58·8±11·9 | } 1·7±22·1 |
| 3–6 „ | 5 | 2 | | |
| 6–12 „ | 7 | 4 | 57·1±18·7 | } 40·5±24·1 |
| Over 12 „ | 6 | 1 | 16·6±15·2 | |
| Not known | 1 | – | | |

The differences in survival rate are not statistically significant.
For cases of known duration $\chi^2=3\cdot32$, $n=2$, $P<\cdot20>\cdot10$.

*Cervix uteri.*

| Duration. | All stages. No. | All stages. Survd. | Per cent. | Difference. |
|---|---|---|---|---|
| 0–6 months | 43 | 24 | 55·8± 7·6 | } 14·6±14·1 |
| 6–12 „ | 17 | 7 | 41·2±11·9 | } 3·2±20·4 |
| Over 12 „ | 9 | 4 | 44·4±16·5 | |
| Not known | 2 | 2 | | |

The differences in survival rate are not statistically significant.
For cases of known duration $\chi^2=1\cdot20$, $n=2$, $P<\cdot70>\cdot50$.

*Bladder—papillary carcinoma.*

| Duration. | All stages. No. | All stages. Survd. | Per cent. | Difference. |
|---|---|---|---|---|
| 0–6 months | 56 | 20 | 35·7± 6·4 | } 2·4±12·1 |
| 6–12 „ | 21 | 7 | 33·3±10·3 | } 12·1±12·5 |
| Over 12 „ | 33 | 7 | 21·2± 7·1 | |
| Not known | 1 | – | | |

The differences in survival rate are not statistically significant.
For cases of known duration $\chi^2=2\cdot12$, $n=2$, $P<\cdot50>\cdot30$.

*Pharynx.*

| Duration. | All stages. No. | All stages. Survd. | Per cent. | Difference. |
|---|---|---|---|---|
| 0–6 months | 14 | 2 | 14·3±9·3 | } 4·3±13·3 |
| 6–12 „ | 4 | – | } 10·0±9·5 | |
| Over 12 „ | 6 | 1 | | |
| Not known | 1 | – | | |

The differences in survival rate are not statistically significant.
For cases of known duration $\chi^2=\cdot102$, $n=1$, $P<\cdot80>\cdot70$.

All cases of cancer of the female breast in Stages I, II and III are included in this table, whether treated by radical mastectomy alone or combined with radiotherapy. The proportions in the three stages are 31·0, 32·6 and 36·4 per cent respectively. Among 383 patients treated by radical mastectomy alone, 35·5 per cent were in Stage I and the best survival rate for all stages combined was 50·9 $\pm$ 3·3 per cent for those treated during the first 6 months; among 175 who were treated by radical mastectomy combined with radiotherapy only 21·1 per cent were in Stage I and 43·4 per cent were in Stage III, and the best survival rate for all stages combined was 58·8 $\pm$ 8·4 per cent for patients treated one year or more subsequent to the first symptom, who numbered 34. The difference of 7·9 $\pm$ 9·0 between these survival rates is not statistically significant.

When the sites were subdivided into those in which the highest survival rates were among the patients who came under treatment in less than 3 months and those in which the highest rates were among those treated in the second 3 months, it was found that the former included squamous carcinoma of the skin, corpus uteri, penis and female breast with a survival rate for all groups of 54·9 per cent, while the latter included cervix uteri, kidney, papillary carcinoma of the bladder and carcinoma of the pharynx (treated by radical surgery or by combined methods) with a 5-year survival rate for all groups of 51·0 per cent.

The age distribution of 702 patients with cancer of the breast who were treated by radical mastectomy alone, of 393 who were treated by radical mastectomy combined with radiotherapy, of 80 patients with cancer of the corpus uteri treated by panhysterectomy and of 95 with cancer of the cervix uteri treated by Wertheim's hysterectomy were investigated by Harnett (1948, 1949); in addition that of 138 patients with squamous carcinoma of the skin and of 40 with carcinoma or hypernephroma of the kidney have been tested by the $\chi^2$ test, but in no case were there any significant differences between the observed and expected survivals in the different age groups.

When the 616 patients who came for treatment within the first 6 months were classified according to the stage of the disease at the time of commencing treatment, it was found that 46·6 per cent were in Stage I, in which the disease is still local, and 53·4 per cent were in later stages with involvement of regional lymph nodes or invasion of adjacent tissues or both.

B. *The highest percentage of 5-year survivals was among the patients who were treated from 6 to 12 months after the first symptom.*

TABLE III.

| Site. | 0–6 months. | | 6–12 months. | | Over 12 months. | | Not known. | |
|---|---|---|---|---|---|---|---|---|
| | No. | Survd. | No. | Survd. | No. | Survd. | No. | Survd. |
| Salivary glands | 5 | 4 | 2 | 2 | 19 | 13 | 1 | – |
| Tonsil | 13 | 3 | 1 | 1 | 4 | 2 | – | – |
| Testis | 19 | 10 | 4 | 3 | 8 | 6 | 1 | 1 |
| Colon, all sites | 187 | 63 | 34 | 12 | 63 | 20 | 13 | 3 |
| Skin, malignant melanoma | 14 | 3 | 9 | 3 | 12 | 2 | 2 | – |
| Nasal sinuses | 25 | 5 | 8 | 2 | 3 | – | 2 | 1 |
| Bladder, infiltrating carcinoma | 38 | 5 | 14 | 3 | 13 | 2 | 2 | – |
| Stomach | 98 | 11 | 62 | 15 | 63 | 9 | 18 | 2 |
| Totals | 399 | 104 | 134 | 41 | 185 | 54 | 39 | 7 |
| Per cent survived | 26·1 | | 30·6 | | 29·2 | | 18·0 | |
| Difference | 4·5 | | | 1·4 | | | | |

Detailed figures of survival by stages are given in Table IV for the colon (all sites) and the stomach ; for the other regions in the group all stages are combined.

### Table IV.

*Colon (all sites).*

| Duration. | Stage I. | | Stage II. | | Stage III. | | Totals. | | Per cent. | Difference. |
|---|---|---|---|---|---|---|---|---|---|---|
| | No. | Survd. | No. | Survd. | No. | Survd. | No. | Survd. | | |
| 0–6 months | 105 | 39 | 35 | 12 | 47 | 12 | 187 | 63 | 33·7±3·4 | 1·6 ±8·9 |
| 6–12 „ | 15 | 6 | 12 | 4 | 7 | 2 | 34 | 12 | 35·3±8·2 | 3·6 ±10·1 |
| Over 12 „ | 31 | 10 | 19 | 5 | 13 | 5 | 63 | 20 | 31·7±5·9 | |
| Not known | 7 | 3 | 3 | – | 3 | – | 13 | 3 | | |

The differences in survival rate are not statistically significant.
For cases of known duration $\chi^2=\cdot 135$, $n=2$, $P<\cdot 95>\cdot 90$.

*Stomach (all sites).*

| Duration. | Stage I. | | Stage II. | | Stage III. | | Totals. | | Per cent. | Difference. |
|---|---|---|---|---|---|---|---|---|---|---|
| | No. | Survd. | No. | Survd. | No. | Survd. | No. | Survd. | | |
| 0–6 months | 38 | 7 | 50 | 2 | 10 | 2 | 98 | 11 | 11·2±3·2 | 13·0 ±6·3 |
| 6–12 „ | 25 | 11 | 30 | 3 | 7 | 1 | 62 | 15 | 24·2±5·4 | 9·9 ±7·0 |
| Over 12 „ | 23 | 5 | 35 | 4 | 5 | – | 63 | 9 | 14·3±4·4 | |
| Not known | 8 | – | 6 | 2 | 4 | – | 18 | 2 | | |

The difference between the survival rates of those operated on within 6 months and those operated on in the second 6 months from the first symptom, 13±6·3, is statistically significant.
For cases of known duration $\chi^2=4\cdot 95$, $n=2$, $P<\cdot 10>\cdot 05$.

*Salivary glands.*

| Duration. | All stages. No. | All stages. Survd. | Per cent. | Difference. |
|---|---|---|---|---|
| 0–6 months | 5 | 4 | 80·0±17·9 | 8·6±20·4 |
| 6–12 „ | 2 | 2 } | 71·4± 9·8 | |
| Over 12 „ | 19 | 13 } | | |
| Not known | 1 | – | | |

The difference is not statistically significant.
For cases of known duration $\chi^2=\cdot 158$, $n=1$, $P<\cdot 70>\cdot 50$.

*Tonsil.*

| Duration. | All stages. No. | All stages. Survd. | Per cent. | Difference. |
|---|---|---|---|---|
| 0–6 months | 13 | 3 | 23·1±11·7 | 36·9±24·8 |
| 6–12 „ | 1 | 1 } | 60·0±21·9 | |
| Over 12 „ | 4 | 2 } | | |

The difference is not statistically significant.
For cases of known duration $\chi^2=2\cdot 23$, $n=1$, $P<\cdot 20>\cdot 10$.

*Testis (excluding cases of teratoma).*

| Duration. | All stages. No. | All stages. Survd. | Per cent. | Difference. |
|---|---|---|---|---|
| 0–6 months | 19 | 10 | 52·6±11·4 | 22·4±17·0 |
| 6–12 „ | 4 | 3 } | 75·0±12·5 | |
| Over 12 „ | 8 | 6 } | | |
| Not known | 1 | 1 | | |

The difference is not statistically significant.
For cases of known duration $\chi^2=1\cdot 56$, $n=1$, $P<\cdot 30>\cdot 20$.

TABLE IV—*continued.*

*Skin—malignant melanoma.*

| Duration. | All stages. No. | All stages. Survd. | Per cent. | Difference. |
|---|---|---|---|---|
| 0–6 months | 14 | 3 | 21·4±10·9 | 11·9±19·2 |
| 6–12 „ | 9 | 3 | 33·3±15·7 | 16·7±19·0 |
| Over 12 „ | 12 | 2 | 16·6±10·7 | |
| Not known | 2 | – | | |

The differences in survival rate are not statistically significant.
For cases of known duration $\chi^2 = \cdot 834$, $n=2$, $P < \cdot 70 > \cdot 50$.

*Bladder—infiltrating carcinoma.*

| Duration. | All stages. No. | All stages. Survd. | Per cent. | Difference. |
|---|---|---|---|---|
| 0–6 months | 38 | 5 | 13·2± 5·5 | 8·2±12·2 |
| 6–12 „ | 14 | 3 | 21·4±10·9 | 6·0±14·8 |
| Over 12 „ | 13 | 2 | 15·4±10·0 | |
| Not known. | 2 | – | | |

The differences in survival rate are not statistically significant.
For cases of known duration $\chi^2 = \cdot 54$, $n=2$, $P < \cdot 80 > \cdot 70$.

*Nasal sinuses.*

| Duration. | All stages. No. | All stages. Survd. | Per cent. | Difference. |
|---|---|---|---|---|
| 0–6 months | 25 | 5 | 20·0± 8·0 | 1·8±14·1 |
| 6–12 „ | 8 | 2 | 18·2±11·6 (6–12 and over 12) | |
| Over 12 „ | 3 | – | | |
| Not known | 2 | 1 | | |

The differences in survival rate are not statistically significant.
For cases of known duration $\chi^2 = \cdot 016$, $n=1$, $P < \cdot 90 > \cdot 80$.

There are no significant differences between the survival rates of the three groups of patients with carcinoma of the colon, nor were any found when the figures for the different regions of the colon were examined separately. In the case of the stomach, however, the best results of operation were in the group of 62 patients treated in the second 6 months from first symptom, whose survival rate was 24·2 per cent against 11·2 per cent for 98 operated on within the first 6 months; the difference of 13·0 ± 6·3 is statistically significant. The group of 63 in whom the symptoms were of over 12 months' standing at the time of operation had a survival rate of 14·3 per cent; the difference of 9·9 ± 7·0 between this figure and that for the group treated in the second 6 months is not statistically significant. If the patients are grouped into those of less than 6 months' duration, with a survival rate of 11·2 per cent, and those of over 6 months' duration, with a survival rate of 19·2 per cent, the difference of 8·0 ± 4·7 is not significant.

The age distribution of the patients with cancer of the stomach and colon were investigated by the $\chi^2$ test, but in neither case were there any significant differences between the observed and expected survivals in the different age groups; for the stomach cases $P < \cdot 20 > \cdot 10$ and for the colon cases $P < \cdot 70 > \cdot 50$, showing that the differences in survival rates were not due to differences in age distribution. The survival rate for cancer of the stomach was 20·4 per cent for 108 patients in the age groups 25–54 and 11·1 per cent for 133 patients of 55 and upwards, but the difference of 9·3 ± 4·7 is not significant, though the prognosis was not so good in the older patients. The 15 survivors of those treated in the second 6 months were distributed evenly over the age-groups, with the greatest number in the 45–54 group.

When the 134 patients who came under treatment 6–12 months from the first symptom and had the best survival rate were classified according to the stage which the disease had reached when treatment was commenced, it was found that 39·6 per cent were still in Stage I and 60·4 per cent were in later stages.

C. *The highest percentage of 5-year survivals was among the patients who were treated 12 months or more subsequent to the first symptom.*

TABLE V.

| Site. | 0–6 months. | | 6–12 months. | | Over 12 months. | | Not known. | |
|---|---|---|---|---|---|---|---|---|
| | No. | Survd. | No. | Survd. | No. | Survd. | No. | Survd. |
| Intrinsic larynx | 8 | 3 | 6 | 2 | 9 | 7 | – | – |
| Thyroid gland | 23 | 12 | 6 | 4 | 20 | 14 | 3 | 3 |
| Vulva and female urethra | 22 | 8 | 14 | 6 | 16 | 11 | 3 | – |
| Lip, upper and lower | 27 | 10 | 10 | 5 | 18 | 12 | 2 | 1 |
| Bone sarcoma (all types) | 27 | 10 | 7 | 4 | 8 | 5 | 1 | – |
| Ovary, malignant papillary cysts | 44 | 16 | 11 | 6 | 12 | 7 | 2 | 1 |
| Sarcoma of soft tissue | 35 | 17 | 4 | 2 | 30 | 17 | 3 | 1 |
| Rectum, ampulla | 137 | 42 | 70 | 22 | 61 | 31 | 11 | – |
| Eye | 32 | 12 | 8 | 4 | 10 | 5 | 7 | 3 |
| Male breast | 3 | – | 2 | 1 | 4 | 2 | – | – |
| Recto-sigmoid | 56 | 15 | 39 | 9 | 25 | 11 | 7 | 3 |
| Tongue | 47 | 15 | 9 | 3 | 14 | 6 | – | – |
| Anal canal and anus | 16 | 5 | 8 | 1 | 8 | 3 | 3 | – |
| Ovary, carcinoma | 37 | 6 | 6 | – | 8 | 3 | 3 | 1 |
| Mouth, all sites | 51 | 8 | 7 | 1 | 13 | 4 | – | – |
| Prostate | 19 | 3 | 8 | 2 | 10 | 3 | 1 | – |
| Totals | 584 | 182 | 215 | 72 | 266 | 141 | 46 | 13 |
| Per cent survived | 31·2 | | 33·5 | | 53·0 | | | |
| Difference | 2·3 | | 19·5 | | | | | |

For the whole group of 1065 cases of known duration the survival rate of 799 patients who were treated within 12 months of the first symptom was 31·8 per cent, whereas for 266 who were treated later it was 53·0 per cent. Detailed figures of survival by stages are given below for the vulva, lip, ovary (malignant papillary cysts), ampulla of rectum, recto-sigmoid, tongue and mouth. For other regions all stages are combined.

TABLE VI.

*Vulva and female urethra.*

| Duration. | Stage I. | | Stage II. | | Stage III. | | Totals. | | Per cent. | Difference. |
|---|---|---|---|---|---|---|---|---|---|---|
| | No. | Survd. | No. | Survd. | No. | Survd. | No. | Survd. | | |
| 0–6 months | 14 | 8 | 1 | – | 7 | – | 22 | 8 | 36·4±10·2 | 6·5 ±16·7 (0–6 vs 6–12) |
| 6–12 „ | 10 | 5 | – | – | 4 | 1 | 14 | 6 | 42·9±13·2 | 25·8 ±17·6 (6–12 vs over 12) |
| Over 12 „ | 11 | 8 | 1 | 1 | 4 | 2 | 16 | 11 | 68·7±11·6 | |
| Not known | – | – | – | – | 3 | – | 3 | – | | |

The differences in survival rate are not statistically significant.
For cases of known duration $\chi^2=4\cdot11$, $n=2$, $P<\cdot20>\cdot10$.

### Table VI—*continued.*

*Lip, upper and lower.*

| Duration. | Stage I. | | Stage II. | | Stage III. | | Totals. | | Per cent. | Difference. |
|---|---|---|---|---|---|---|---|---|---|---|
| | No. | Survd. | No. | Survd. | No. | Survd. | No. | Survd. | | |
| 0–6 months | 16 | 8 | 1 | – | 10 | 2 | 27 | 10 | 37·0± 9·3 | 13·0 |
| 6–12 „ | 4 | 2 | – | – | 6 | 3 | 10 | 5 | 50·0±15·8 | ±18·3<br>16·6 |
| Over 12 „ | 14 | 11 | 1 | – | 3 | 1 | 18 | 12 | 66·6±11·1 | ±19·3 |
| Not known | 2 | 1 | – | – | – | – | 2 | 1 | | |

The differences in survival rate are not statistically significant.
For cases of known duration $\chi^2=3\cdot794$, $n=2$, $P<\cdot20>\cdot10$.

*Ovary—malignant papillary cysts.*

| Duration. | Stage I. | | Stage II. | | Stage III. | | Totals. | | Per cent. | Difference. |
|---|---|---|---|---|---|---|---|---|---|---|
| | No. | Survd. | No. | Survd. | No. | Survd. | No. | Survd. | | |
| 0–6 months | 36 | 15 | 8 | 1 | – | – | 44 | 16 | 36·4± 7·2 | 18·1 |
| 6–12 „ | 9 | 5 | 2 | 1 | – | – | 11 | 6 | 54·5±15·0 | ±16·7<br>3·8 |
| Over 12 „ | 10 | 7 | 2 | – | – | – | 12 | 7 | 58·3±14·2 | ±20·7 |
| Not known | 2 | 1 | – | – | – | – | 2 | 1 | | |

The differences in survival rate are not statistically significant.
For cases of known duration $\chi^2=2\cdot56$, $n=2$, $P<\cdot30>\cdot20$.

*Rectum, ampulla.*

| Duration. | Stage I. | | Stage II. | | Stage III. | | Totals. | | Per cent. | Difference. |
|---|---|---|---|---|---|---|---|---|---|---|
| | No. | Survd. | No. | Survd. | No. | Survd. | No. | Survd. | | |
| 0–6 months | 40 | 19 | 44 | 14 | 53 | 9 | 137 | 42 | 30·7±3·9 | 0·7 |
| 6–12 „ | 22 | 9 | 25 | 8 | 23 | 5 | 70 | 22 | 31·4±5·6 | ±6·8<br>19·4 |
| Over 12 „ | 16 | 11 | 23 | 11 | 22 | 9 | 61 | 31 | 50·8±6·4 | ±8·5 |
| Not known | 6 | – | 3 | – | 2 | – | 11 | – | | |

The difference of 19·4±8·5 per cent between the survival rate of those patients who were treated in the second 6 months and of those whose duration was more than 12 months is statistically significant.

For cases of known duration $\chi^2=8\cdot17$, $n=2$, $P<\cdot02>\cdot01$.

*Recto-sigmoid.*

| Duration. | Stage I. | | Stage II. | | Stage III. | | Totals. | | Per cent. | Difference. |
|---|---|---|---|---|---|---|---|---|---|---|
| | No. | Survd. | No. | Survd. | No. | Survd. | No. | Survd. | | |
| 0–6 months | 15 | 3 | 17 | 6 | 24 | 6 | 56 | 15 | 26·8±5·9 | 3·7 |
| 6–12 „ | 13 | 4 | 10 | 4 | 16 | 1 | 39 | 9 | 23·1±6·7 | ±9·0<br>20·9 |
| Over 12 „ | 9 | 6 | 8 | 4 | 8 | 1 | 25 | 11 | 44·0±9·9 | ±12·0 |
| Not known | 3 | 2 | 4 | 1 | – | – | 7 | 3 | | |

The differences in survival rate are not statistically significant.
For cases of known duration $\chi^2=3\cdot52$, $n=2$, $P<\cdot20>\cdot10$.

TABLE VI—*continued.*

*Tongue.*

| Duration. | Stage I. No. | Stage I. Survd. | Stage II. No. | Stage II. Survd. | Stage III. No. | Stage III. Survd. | Totals. No. | Totals. Survd. | Per cent. | Difference. |
|---|---|---|---|---|---|---|---|---|---|---|
| 0–6 months | 29 | 12 | 2 | 1 | 16 | 2 | 47 | 15 | 31·9±6·8 | |
| 6–12 „ | 7 | 3 | – | – | 2 | – | 9 | 3 | 33·3±15·7 | 1·4 ±17·1 |
| Over 12 „ | 9 | 5 | – | – | 5 | 1 | 14 | 6 | 42·9±13·2 | 9·6 ±20·5 |

The differences in survival rate are not statistically significant.
For cases of known duration $\chi^2 = \cdot 585$, $n=2$, $P < \cdot 80 > \cdot 70$.

*Mouth—all sites.*

| Duration | Stage I. No. | Stage I. Survd. | Stage II. No. | Stage II. Survd. | Stage III. No. | Stage III. Survd. | Totals. No. | Totals. Survd. | Per cent. | Difference. |
|---|---|---|---|---|---|---|---|---|---|---|
| 0–6 months | 21 | 5 | 10 | 1 | 20 | 2 | 51 | 8 | 15·7± 5·1 | |
| 6–12 „ | 3 | 1 | – | – | 4 | – | 7 | 1 | 14·3±13·2 | 1·4 ±14·2 |
| Over 12 „ | 9 | 5 | – | – | 5 | 1 | 14 | 6 | 42·9±13·2 | 28·6 ±18·7 |

The differences in survival rate are not statistically significant.
For cases of known duration $\chi^2 = 1 \cdot 65$, $n=2$, $P < \cdot 50 > \cdot 30$.

*Intrinsic larynx.*

| Duration. | All stages. No. | All stages. Survd. | Per cent. | Difference. |
|---|---|---|---|---|
| 0–6 months | 8 | 3 | 37·5±17·1 | |
| 6–12 „ | 6 | 2 | 33·3±19·2 | 4·2±25·7 |
| Over 12 „ | 9 | 7 | 77·7±13·9 | 44·4±23·7 |

The differences in survival rate are not statistically significant.
For cases of known duration $\chi^2 = 3 \cdot 92$, $n=2$, $P < \cdot 20 > \cdot 10$.

*Thyroid Gland,*

| Duration. | All stages. No. | All stages. Survd. | Per cent. | Difference. |
|---|---|---|---|---|
| 0–6 months | 23 | 12 | 52·2±10·4 | |
| 6–12 „ | 6 | 4 | 66·6±19·2 | 14·4±21·9 |
| Over 12 „ | 20 | 14 | 70·0±10·2 | 3·4±21·8 |
| Not known | 3 | 3 | | |

The differences in survival rate are not statistically significant.
For cases of known duration $\chi^2 = 1 \cdot 55$, $n=2$, $P < \cdot 50 > \cdot 30$. If the 3 cases of unknown duration are included $\chi^2 = 3 \cdot 505$, $n=3$, $P < \cdot 50 > \cdot 30$.

*Bone sarcoma (all types).*

| Duration. | All stages. No. | All stages. Survd. | Per cent. | Difference. |
|---|---|---|---|---|
| 0–6 months | 27 | 10 | 37·0± 9·3 | |
| 6–12 „ | 7 | 4 | 57·1±18·7 | 20·1±20·9 |
| Over 12 „ | 8 | 5 | 62·5±17·1 | 5·4±25·3 |
| Not known | 1 | – | | |

The differences in survival rate are not statistically significant.
For cases of known duration $\chi^2 = 2 \cdot 09$, $n=2$, $P < \cdot 50 > \cdot 30$.

TABLE VI—*continued.*

*Soft tissue sarcoma (all types).*

| Duration. | All stages. No. | All stages. Survd. | Per cent. | Difference. |
|---|---|---|---|---|
| 0–6 months | 35 | 17 | | |
| 6–12 ,, | 4 | 2 | 48·7±8·0 | 7·9±12·1 |
| Over 12 ,, | 30 | 17 | 56·6±9·0 | |
| Not known | 3 | 1 | | |

The difference in survival rate is not statistically significant.
For cases of known duration $\chi^2 = \cdot 44$, $n = 1$, $P < \cdot 70 > \cdot 50$.

*Eye (all types of malignant growth).*

| Duration. | All stages. No. | All stages. Survd. | Per cent. | Difference. |
|---|---|---|---|---|
| 0–6 months | 32 | 12 | 37·5± 8·5 | 12·5±19·6 |
| 6–12 ,, | 8 | 4 | 50·0±17·7 | 0·0 |
| Over 12 ,, | 10 | 5 | 50·0±15·8 | |
| Not known | 7 | 3 | | |

The difference in survival rate is not statistically significant.
For cases of known duration $\chi^2 = \cdot 73$, $n = 2$, $P < \cdot 70 > \cdot 50$.

*Anal canal and anus.*

| Duration. | All stages. No. | All stages. Survd. | Per cent. | Difference. |
|---|---|---|---|---|
| 0–6 months | 16 | 5 | 31·2±11·6 | 18·7±16·4 |
| 6–12 ,, | 8 | 1 | 12·5±11·7 | 25·0±20·7 |
| Over 12 ,, | 8 | 3 | 37·5±17·1 | |
| Not known | 2 | – | | |

The differences in survival rate are not statistically significant.
For cases of known duration $\chi^2 = 1 \cdot 39$, $n = 2$, $P < \cdot 50 > \cdot 30$.

*Ovary—carcinoma and sarcoma.*

| Duration. | All stages. No. | All stages. Survd. | Per cent. | Difference. |
|---|---|---|---|---|
| 0–6 months | 37 | 6 | 16·2± 6·0 | |
| 6–12 ,, | 6 | – | 0·0 | 21·3±18·1 |
| Over 12 ,, | 8 | 3 | 37·5±17·1 | |
| Not known | 3 | 1 | | |

The difference in survival rate is not statistically significant.
For cases of known duration $\chi^2 = 3 \cdot 51$, $n = 2$, $P < \cdot 20 > \cdot 10$.

*Prostate.*

| Duration. | All stages. No. | All stages. Survd. | Per cent. | Difference. |
|---|---|---|---|---|
| 0–6 months | 19 | 3 | 15·8± 8·4 | 9·2±17·4 |
| 6–12 ,, | 8 | 2 | 25·0±15·3 | 5·0±21·0 |
| Over 12 ,, | 10 | 3 | 30·0±14·5 | |
| Not known | 1 | – | | |

The differences in survival rate are not statistically significant.
For cases of known duration $\chi^2 = \cdot 85$, $n = 2$, $P < \cdot 70 > \cdot 50$.

In all these sites the 5-year survival rates of those patients who came under treatment 12 months or more after the first symptom were better than those of the patients who were treated at an earlier stage, but the only difference which is statistically significant is between the survival rate of 50·8 per cent for 61

patients with cancer of the ampulla of the rectum of over 12 months' standing and 31·4 per cent for 70 patients whose disease was of 6–12 months' standing, difference 19·4 $\pm$ 8·3. When the survival rates for cancer of the ampulla of the rectum were tested by the $\chi^2$ test the value of $P < \cdot 02 > \cdot 01$ was found, mainly due to the difference between the observed and expected survivals in the group of over 12 months' duration.

The age distribution of the patients with cancer of the recto-sigmoid and of those with cancer of the ampulla of the rectum were investigated by the $\chi^2$ test, but in neither case were any significant differences found between the observed and expected survivals; for both regions $P < \cdot 90 > \cdot 80$, showing that the differences in survival rates were not due to differences in age distribution.

When the 266 patients who came under treatment more than 12 months from the first symptom and had the highest survival rate were classified according to the stage of the disease at the time of commencing treatment, it was found that 53·8 per cent were still in Stage I and 46·2 were in later stages, a higher percentage of Stage I cases than in either Group A or Group B.

In Table VII all three groups are combined:

TABLE VII.

| Group. | Duration. | 0–6 months. No. | 0–6 months. Survd. | 6–12 months. No. | 6–12 months. Survd. | Over 12 months. No. | Over 12 months. Survd. | Not known. No. | Not known. Survd. |
|---|---|---|---|---|---|---|---|---|---|
| A | 0–6 months | 616 | 315 | 211 | 83 | 270 | 119 | 48 | 15 |
| B | 6–12 „ | 399 | 104 | 134 | 41 | 185 | 54 | 39 | 7 |
| C | Over 12 „ | 584 | 182 | 215 | 72 | 266 | 141 | 46 | 13 |
| | Totals | 1599 | 601 | 560 | 196 | 721 | 314 | 133 | 35 |
| | Per cent survived | 37·6 | | 35·0 | | 43·6 | | 26·3 | |
| | Difference | 2·6 | | 8·6 | | | | | |

TABLE VIII.—*Grand Totals of Cases of Known Duration.*

| Duration. | No. | Survived. | Per cent. | Difference. |
|---|---|---|---|---|
| Under 12 months | 2159 | 797 | 36·9 | 6·7 |
| Over „ | 721 | 314 | 43·6 | |

The difference of survival rate between those who were treated within 6 months of the first symptom and those who were treated 6–12 months from the first symptom is small, but the survival rate of those who were treated after a delay of 12 months or more is greater than that of the other two groups, either combined or separately; numerically the latter group comprises about one-quarter of all cases. As has been stated above this difference cannot be accounted for by differences in the age distribution of patients in either of the groups, so it must be presumed to be due to variations in the rate of growth of individual tumours and in their infiltrating and metastatising powers. All cases of patients with distant metastases have been eliminated from the present series, which was confined to those in which the tumour was local or had given rise to clinically recognisable metastases in lymph nodes; they were selected as operable cases, no patient in an advanced stage or suitable only for palliative treatment being included. Small differences were found in the proportion of patients in Stage I among those with the highest survival rate in each of the three groups; for Group

A this was 46·6 per cent, for Group B it was 39·6 per cent and for Group C it was 53·8 per cent, but the combined figures for all the patients in each group showed only small deviations from the mean.

*Classification by Stages of all Cases of Known Duration.*

TABLE IX.

| Stage. | A. 0–6 months. No. | Per cent. | B. 6–12 months. No. | Per cent. | C. Over 12 months. No. | Per cent. | Totals. No. | Per cent. |
|---|---|---|---|---|---|---|---|---|
| I | 526 | 47·9 | 323 | 45·0 | 550 | 51·6 | 1399 | 48·6 |
| II | 315 | 28·7 | 256 | 35·7 | 234 | 22·0 | 805 | 28·0 |
| III | 256 | 23·3 | 139 | 19·4 | 281 | 26·4 | 676 | 23·5 |
| Totals | 1097 | 99·9 | 718 | 100·1 | 1065 | 100·0 | 2880 | 100·1 |

The highest percentage of Stage I cases is among the patients whose disease was of over 12 months' standing (Group C), probably due, like the difference in survival rates between those treated early and those treated later, to variations in the intrinsic growth-rate of the tumours themselves. Park and Lees (1951) point out that this may be estimated by (*a*) the histological appearance of the tumour, disregarding size or degree of extension at the time of diagnosis, or (*b*) by duration of survival from the onset of symptoms or time of treatment to death in untreated or unsuccessfully treated cases, though in the latter case the survival time may be affected by the treatment.

*Pathology.*

Histological reports were available in 88 per cent of the 3013 cases. In 376 cases the histological grade of the growths according to Broders' classification was given, but as these reports were the work of several different pathologists the system of grading used may not have been uniform ; moreover the cases in which these particulars were given were so few that there were rarely enough in any one site to provide material for an analysis. For all sites combined there were 134 survivors of the 376 patients (35·6 per cent) graded as shown in Table X.

TABLE X.

| Grade. | No. | Survived. | Per cent. |
|---|---|---|---|
| I | 83 | 40 | 48·2 |
| II | 153 | 57 | 37·3 |
| III | 66 | 17 | 25·8 |
| IV | 74 | 20 | 27·0 |

Table XI shows the 376 cases arranged according to grade and duration of symptoms prior to treatment.

TABLE XI.

| Grade. | 0–6 months. | 6–12 months. | Over 12 months. | Not known. | Totals. |
|---|---|---|---|---|---|
| I | 36 | 21 | 23 | 3 | 83 |
| II | 74 | 39 | 34 | 6 | 153 |
| III | 40 | 8 | 15 | 3 | 66 |
| IV | 39 | 22 | 10 | 3 | 74 |
| Totals | 189 | 90 | 82 | 15 | 376 |
| Percentage in Grades I and II | 58·2 | 66·6 | 69·5 | 60·0 | 62·8 |

TABLE XI—*continued*

*Rectum and recto-sigmoid*—117 cases.

| Grade. | 0–6 months. | 6–12 months. | Over 12 months. | Not known. | Totals. |
|---|---|---|---|---|---|
| I | 2 | 4 | 2 | 1 | 9 |
| II | 34 | 23 | 19 | 1 | 77 |
| III | 15 | 4 | 3 | 3 | 25 |
| IV | 2 | 3 | 1 | – | 6 |
| Totals | 53 | 34 | 25 | 5 | 117 |
| Percentage in Grades I and II | 68·0 | 79·4 | 84·0 | 40·0 | 73·5 |

The differences are not significant. $\chi^2 = 2 \cdot 84$, $n = 2$, $P < \cdot 30 > \cdot 20$.

*Stomach*—35 cases.

| Grade. | 0–6 months. | 6–12 months. | Over 12 months. | Not known. | Totals. |
|---|---|---|---|---|---|
| I | 4 | 3 | 3 | 1 | 11 |
| II | 1 | 2 | 1 | 1 | 5 |
| III | 2 | – | 2 | – | 4 |
| IV | 9 | 2 | 4 | – | 15 |
| Totals | 16 | 7 | 10 | 2 | 35 |
| Percentage in Grades I and II | 31·2 | 71·4 | 40·0 | 100·0 | 45·7 |

The differences are not significant, but it has been shown in an earlier section that the highest survival rate among patients with cancer of the stomach was among those in whom the delay in treatment was 6–12 months. $\chi^2 = 3 \cdot 47$, $n = 2$, $P < \cdot 20 > \cdot 10$.

Scanty as these data are, they are in accord with Bloom's (1950*b*) findings that a higher percentage of patients with tumours of Grades III and IV come early for treatment than of those with the lower grades.

*Duration of survival of untreated patients.*

Many patients were not treated for various reasons, and the total duration of the disease from first sympton to death is known in most of these. Patients who were in Stage IV of the disease with distant metastases have not been included in the following list, in which the sites are arranged in order of mean duration of survival of patients in Stages I, II and III in whose cases treatment was withheld for other reasons than the advanced stage of the disease (Table XII). The actual duration of survival expressed in months is given, and also expressed as a percentage of the normal expectation of life during the next 5 years for a group of persons having the same sex-age distribution as the group of cancer patients under consideration (Table XII).

The last column of Table XII refers to the three groups into which the sites were divided according to the survival rates in relation to delay in commencing treatment; the figures show that there is no correspondence between these groups and the duration of life in the untreated cases. The mean duration of life in 153 untreated patients in Group A was 23·1 months, of 176 in Group B was 11·7 months and of 296 in Group C was 17·6 months. The sites showing the longest survivals among the untreated patients are those in which treatment by radical surgery alone or with radiotherapy gives the best results, although some sites show a short duration of life, either because, as in the case of the lip, very few cases are left untreated, or because, as in the case of the colon and the intrinsic larynx, untreated patients often die of complications, such as intestinal

TABLE XII.

| Site. | Number of patients. | Actual duration in months. | Percentage of normal expectation. | Group. |
|---|---|---|---|---|
| Breast | 53 | 35·9 | 72·8 | A |
| Skin, squamous carcinoma | 7 | 34·9 | 74·6 | A |
| Recto-sigmoid | 29 | 22·4 | 43·9 | C |
| Bladder, papillary carcinoma | 23 | 22·0 | 43·3 | A |
| Anus and anal canal | 14 | 21·9 | 44·5 | C |
| Prostate | 43 | 19·5 | 39·2 | C |
| Vulva | 9 | 19·1 | 40·0 | C |
| Rectum, ampulla | 132 | 19·0 | 38·5 | C |
| Cervix uteri | 26 | 17·5 | 31·6 | A |
| Corpus uteri | 7 | 17·4 | 32·9 | A |
| Thyroid gland | 8 | 15·6 | 29·1 | C |
| Bladder, infiltrating carcinoma | 80 | 14·0 | 27·0 | B |
| Nasal sinuses | 11 | 13·0 | 25·2 | B |
| Mouth, floor of | 11 | 12·6 | 24·7 | C |
| Stomach, pylorus | 27 | 11·5 | 22·1 | B |
| Mouth, alveolus | 5 | 10·8 | 22·2 | C |
| „ palate | 8 | 10·0 | 20·9 | C |
| Lip, upper (1), lower (2) | 3 | 10·0 | 22·8 | C |
| Ovary, carcinoma | 5 | 9·6 | 18·0 | C |
| Tongue | 14 | 9·1 | 17·2 | C |
| Intrinsic larynx | 15 | 9·0 | 17·9 | C |
| Tonsil | 7 | 8·7 | 18·5 | B |
| Colon (all sites) | 31 | 8·7 | 17·4 | B |
| Stomach, mid-gastric | 12 | 8·7 | 17·6 | B |
| Pharynx | 28 | 8·4 | 16·3 | A |
| Kidney | 9 | 7·7 | 13·8 | A |
| Stomach, cardiac | 8 | 5·7 | 11·3 | B |

obstruction or broncho-pneumonia comparatively early in the growth of the tumour. In some sites, for example salivary glands, malignant melanoma of skin, penis, testis, sarcoma of soft tissue and of bone, all patients were treated except those whose disease was in Stage IV. The remaining sites in which the duration of survival of untreated patients was less than 12 months—mouth, ovary (carcinoma), tongue, tonsil, stomach and pharynx—are sites in which the results of radical surgery are not so good as in those in which the natural duration of the disease is longer. The conclusion to be drawn is that the growth rate of the tumour is the most important factor influencing the survival rate after radical surgical treatment, leading to relatively long survival in spite of delay in commencing treatment.

SUMMARY.

1. The 5-year survival rates of 2880 patients, whose disease was in Stages I, II and III and who were treated by radical surgery alone or with radiotherapy, were analyzed to ascertain whether the interval between first symptom and commencement of treatment was directly related to the survival rate.

2. It was found that the sites of the disease could be divided into three groups: A. Those in which the highest percentage of 5-year survivals was among the patients treated within the first 6 months from first symptom. B. Those in which it was among the patients treated 6–12 months from first symptom. C. Those in which it was among the patients treated 12 months or more subsequent to the first symptom.

3. Group A included the skin (squamous carcinoma), corpus uteri, penis, cervix uteri, female breast, kidney, bladder (papillary carcinoma) and pharynx. For this group of sites, comprising 1097 cases of known duration, the best survival rate was 51·1 per cent of 616 patients treated in the first 6 months against 42·0 per cent for the remainder. The differences in survival rate were calculated for individual sites, but only those for the skin and one of the differences for the kidney were found to be statistically significant.

4. Group B included salivary glands, tonsil, testis, colon, skin (malignant melanoma), nasal sinuses, bladder (infiltrating carcinoma) and stomach. For this group of sites, comprising 718 cases of known duration, the best survival rate was 30·6 per cent of 134 patients treated in the second 6 months against 27·1 per cent for the remainder. None of the differences in survival rate for individual sites was significant, except that for carcinoma of stomach (all sites) of duration 0–6 months and that for duration 6–12 months, which was 13·0 $\pm$ 6·3 per cent. For all 223 cases of carcinoma of stomach of known duration the survival rates of the cases of 0–6, 6–12 and over 12 months' interval groups tested by the $\chi^2$ test gives the value of $P < \cdot 10 > \cdot 05$ for the difference between observed and expected survivals, which is not statistically significant.

5. Group C included intrinsic larynx, thyroid gland, vulva, lip, bone sarcoma and soft tissue sarcoma, ovary, rectum (ampulla), recto-sigmoid, eye, male breast, anal canal and anus, tongue, mouth and prostate. For this group of 1065 cases of known duration the best survival rate was 53·0 per cent of 266 patients treated more than 12 months from the first symptom against 31·8 per cent of 799 patients treated earlier. In individual sites the only difference which was statistically significant was that between the survival rate of cases of carcinoma of the ampulla of the rectum treated within 12 months of onset, 31·4 per cent and those treated later, 50·8 per cent, difference 19·4 $\pm$ 8·5.

6. The age distribution of the patients was investigated for the most important sites, but in none was any significant difference found between observed and expected survivals due to the differences in age distribution.

7. The highest percentage of patients whose disease was still in Stage I—51·6 per cent—was in Group C, in which the best survival rate was among the patients treated more than 12 months subsequent to the first symptom. It was concluded that the differences in survival rate were probably due to variations in the intrinsic growth rate of the tumours.

8. Only comparatively few observations on the grading of the tumours according to Broders' system were available, but those for carcinoma of the stomach and rectum supported the view that patients with slow-growing tumours formed a high percentage of those who delayed longest before coming for treatment.

9. The total duration of the disease was calculated for 625 patients in Stages I, II and III who were not treated. It was found that the sites showing the longest survivals of untreated patients were those in which the results of radical surgery were most satisfactory.

My thanks are due to the British Empire Cancer Campaign for permission to make use of the records of cases of cancer in London hospitals collected by the Clinical Cancer Research Committee of the Campaign, and to Dr. Percy Stocks for his invaluable advice and help in the statistical treatment of the data.

### REFERENCES.

Balfour, D. C.—(1937) *Ann. Surg.*, **105**, 733.
Bloom, H. J. G.—(1950*a*) *Brit. J. Cancer*, **4**, 259.—(1950*b*) *Ibid.*, **4**, 347.
*British Medical Journal.*—(1952) i, 313.
Eggers, C. de Cholnoky, T., and Jessop, D. S. D.—(1941) *Ann. Surg.*, **113**, 321.
Harnett, W. L.—(1948) *Brit. J. Cancer*, **2**, 212.—(1949) *Ibid.*, **3**, 433.—(1952) 'A Survey of Cancer in London.' Brit. Emp. Cancer Campaign.
Macdonald, I.—(1942) *Surg. Gynec. Obstet.*, **74**, 75.
Park, W. W., and Lees, J. C.—(1951) *Ibid.*, **93**, 129.
Swynnerton, B. F., and Truelove, S. C.—(1952) *Brit. med. J.*, i, 287.
Walters, W., Gray, H. K., and Priestley, J. T.—(1942) 'Carcinoma and other Malignant Lesions of the Stomach.' Philadelphia and London (Saunders).